# What mechanisms mediate prior probability effects on rapid-choice decision-making?

**DOI:** 10.1371/journal.pone.0288085

**Published:** 2023-07-07

**Authors:** Rohan Puri, Mark R. Hinder, Andrew Heathcote

**Affiliations:** 1 Sensorimotor Neuroscience and Ageing Research Group, School of Psychological Sciences, College of Health and Medicine, University of Tasmania, Hobart, Australia; 2 School of Psychology, University of Newcastle, Newcastle, Australia; 3 Department of Psychology, University of Amsterdam, Amsterdam, The Netherlands; Federal University of Paraiba, BRAZIL

## Abstract

Rapid-choice decision-making is biased by *prior probability* of response alternatives. Conventionally, prior probability effects are assumed to *selectively* affect, response threshold, which determines the amount of evidence required to trigger a decision. However, there may also be effects on the rate at which evidence is accumulated and the time required for non-decision processes (e.g., response production). Healthy young (*n* = 21) and older (*n* = 20) adults completed a choice response-time task requiring left- or right-hand responses to imperative stimuli. Prior probability was manipulated using a warning stimulus that informed participants that a particular response was 70% likely (i.e., the imperative stimulus was either congruent or incongruent with the warning stimulus). In addition, prior probability was either fixed for blocks of trials (block-wise bias) or varied from trial-to-trial (trial-wise bias). Response time and accuracy data were analysed using the racing diffusion evidence-accumulation model to test the selective influence assumption. Response times for correct responses were slower on incongruent than congruent trials, and older adults’ responses were slower, but more accurate, than young adults. Evidence-accumulation modelling favoured an effect of prior probability on both response thresholds *and* nondecision time. Overall, the current results cast doubt on the selective threshold influence assumption in the racing diffusion model.

## Introduction

Humans live in a dynamic, ever-changing, world requiring navigation, in a timely manner, through different possible decision outcomes to achieve goal-directed behaviour. Often, these possible decision outcomes–conceptualised here as the time-dependent accumulation of sensory evidence towards a response threshold–are influenced by factors such as different payoffs or prior probabilities [1]. For example, when getting ready to return a serve in tennis, a player may weigh a possible forehand return more than a backhand return based on the known higher prior probability of the opponent serving to their forehand. Then, if the opponent does indeed serve to the forehand, the player would have a quicker reaction-time (and thus higher chance of making a good return) than if the opponent unexpectedly served to their backhand. This effect of prior probability on decision-making processes is classically thought to *selectively* influence response thresholds, such that the more likely outcome has a lower response threshold, and the less likely outcome has a higher response threshold. In the previous example, the response threshold for a forehand return would be lower than that for a backhand return, thus facilitating a faster reaction-time. This canonical effect of prior probability on response thresholds has been observed in domains of perceptual decision-making [2–9] as well as recognition memory [8, 10, 11].

Recently, however, the selective influence of prior probability on response thresholds has come under question. Specifically, it has been claimed that prior probability can also bias sensory evidence [9, 12]. Given this potential non-selectivity of prior probability on decision processes, one may also wonder if, and how, prior probability affects *nondecision*-based processes such as stimulus encoding and response execution. Indeed, mediation by more than one mechanism occurs for another canonical finding in decision-making research, the ‘speed-accuracy trade-off’. The trade-off is named as such because participants are often quicker, but less accurate, when responding under instructions or feedback/payoffs that emphasise speed, and are slower, but more accurate, when responding under instructions or feedback/payoffs that emphasise accuracy. The effect of the speed-accuracy trade-off on decision-based processes was thought to be selectively reflected in response *caution*, mediated by an increase in thresholds for all responses, such that participants are more cautious when responding with an accuracy, rather than speed, emphasis [2, 13, 14]. However, in recent times, it has been demonstrated that the effect of the speed-accuracy trade-off is non-selective. Specifically, this non-selectivity of decision-based processes is observed in the quality of sensory evidence [15–22] and, relevant to the current study, in nondecision-based processes as well, with speed emphasis reducing the time for these processes to be executed [23–25].

Accordingly, given the potential non-selectivity of prior probability on decision-based processes and the effect of other classical manipulations on nondecision-based processes, the primary aim of the current study was to investigate the effect of prior probability on nondecision-based processes. To assess the generality of any such effects, our study incorporated two further manipulations. The first pertains to the *flexibility* with which prior probability information is utilised. Specifically, the more probable response was either kept constant across a block of trials (‘block-wise bias’; [2, 3, 5, 7, 8, 10, 11]) or varied from trial-to-trial (‘trial-wise bias’; [4, 9]). Recently, a direct comparison between these two biasing procedures revealed that effects on response thresholds were greater for block-wise compared to trial-wise biasing [26]. Given this effect on decision-based processes, the current study aimed to elucidate whether any effect of prior probability on nondecision-based processes also differed between block- and trial-wise biasing procedures. The second is a manipulation that addresses the influence of healthy ageing on the utilisation of prior probability information. Garton and colleagues [26] demonstrated that older adults differ in their use of prior probability information and change their thresholds to a greater degree than young adults, at least with a block-wise bias manipulation. Given this effect on decision-based processes, the age-related decline in motor function [27], and the known difference in nondecision-based processing between young and older adults [28], the current study aimed to elucidate whether any effect of prior probability on nondecision-based processes varied due to healthy ageing as well as its interaction with the two, aforementioned, biasing procedures.

**Fig 1:**
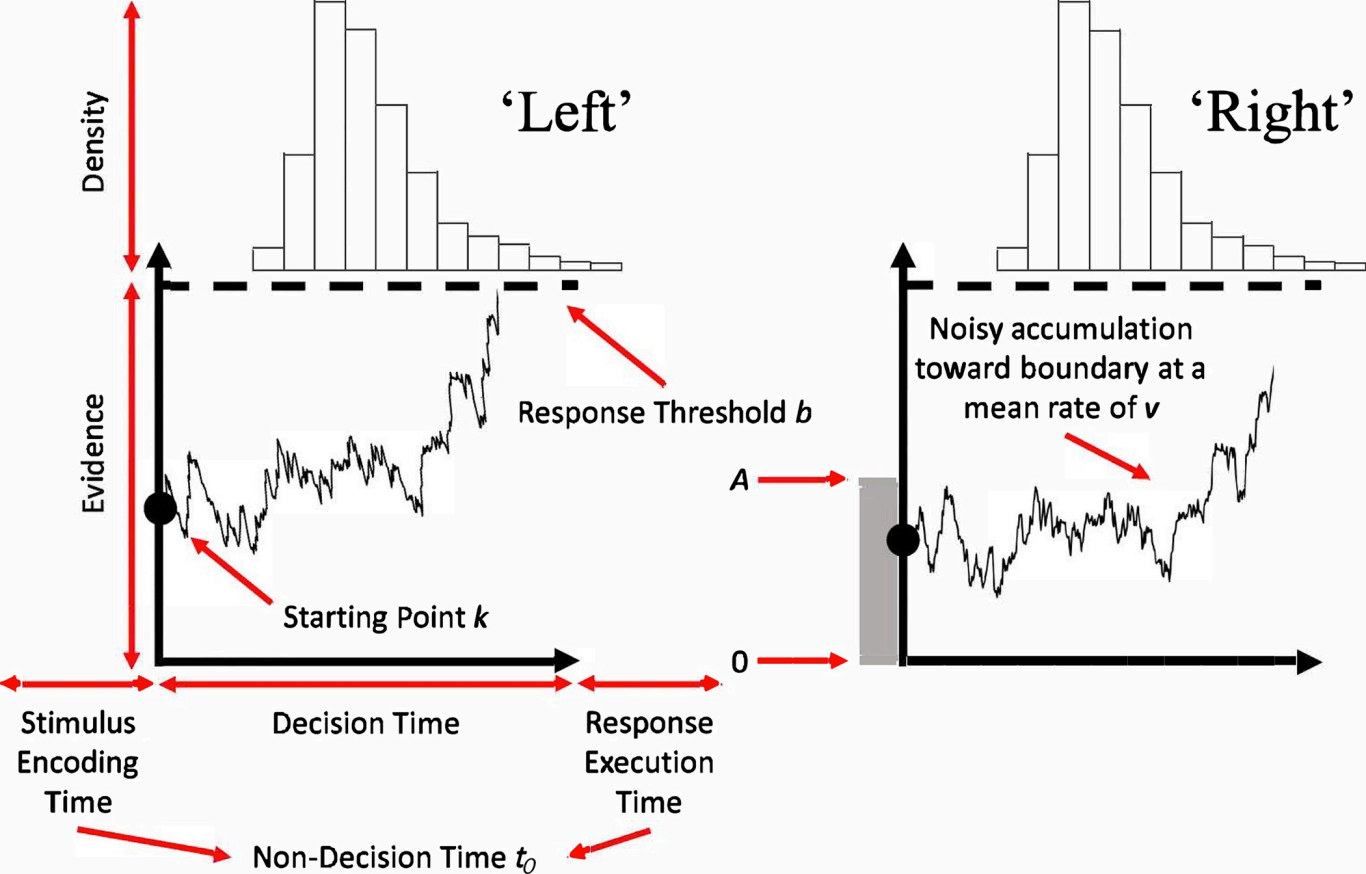
Racing diffusion model. Two-choice model representing a decision between left and right responses. Each accumulator begins with an initial amount of evidence (*k*), drawn from a uniform distribution [0, *A*], that increases at a noisy rate, with mean *v*, towards the response threshold (*b*). Decision time–the time taken for the winning accumulator (in this example, the Left response) to reach its respective threshold, along with nondecision time (*t*_*0*_)–time taken for stimulus encoding and response execution components–determine the overall response time. Adapted with permission (not part of governing open access license) from Springer Nature Customer Service Centre GmbH: Springer Nature, Psychonomic Bulletin & Review, Tillman, G., Zandt, T. Van, & Logan, G. D. Sequential sampling models without random between-trial variability: the racing diffusion model of speeded decision making, *The Psychonomic Soceity*, *Inc*. *(2020)*.

To this end, in the current study of binary choice, the evidence-accumulation process for speeded decisions was characterised using a “sequential sampling model”, namely the *racing diffusion model* [29, 30]. Specifically, as illustrated in Fig 1, each accumulator–representing a decision associated with a ‘left’ or ‘right’ response–begins with an initial amount of evidence and accumulates further evidence in a noisy manner. This process of evidence-accumulation stops when the first accumulator reaches its threshold, with RT corresponding to the time taken to do so (decision time) in addition to stimulus encoding and response execution time (i.e., nondecision time). In the current study, our use of this racing diffusion model makes two key assumptions. Firstly, it was reasoned that (any) effects of prior probability on the nondecision time parameter was reflective of effects on the response execution time, and not the stimulus encoding time, component. Given the identical optical properties of the stimuli in the current study (see ‘Experimental procedure’ section) and that stimulus encoding extracts decision-relevant evidence, it is highly unlikely that the stimulus encoding time component is different for the left vs. right accumulators. In contrast, differential priming of left vs. right responses based on prior probability is highly plausible, resulting in differences in the response execution time component. Indeed, based on transcranial magnetic stimulation studies using electromyography, it has been demonstrated that the corticospinal tract projecting to the effector corresponding to the higher probability response exhibits greater excitation and reduced inhibition than the effector corresponding to the lower probability response [31, 32].

**Fig 2:**
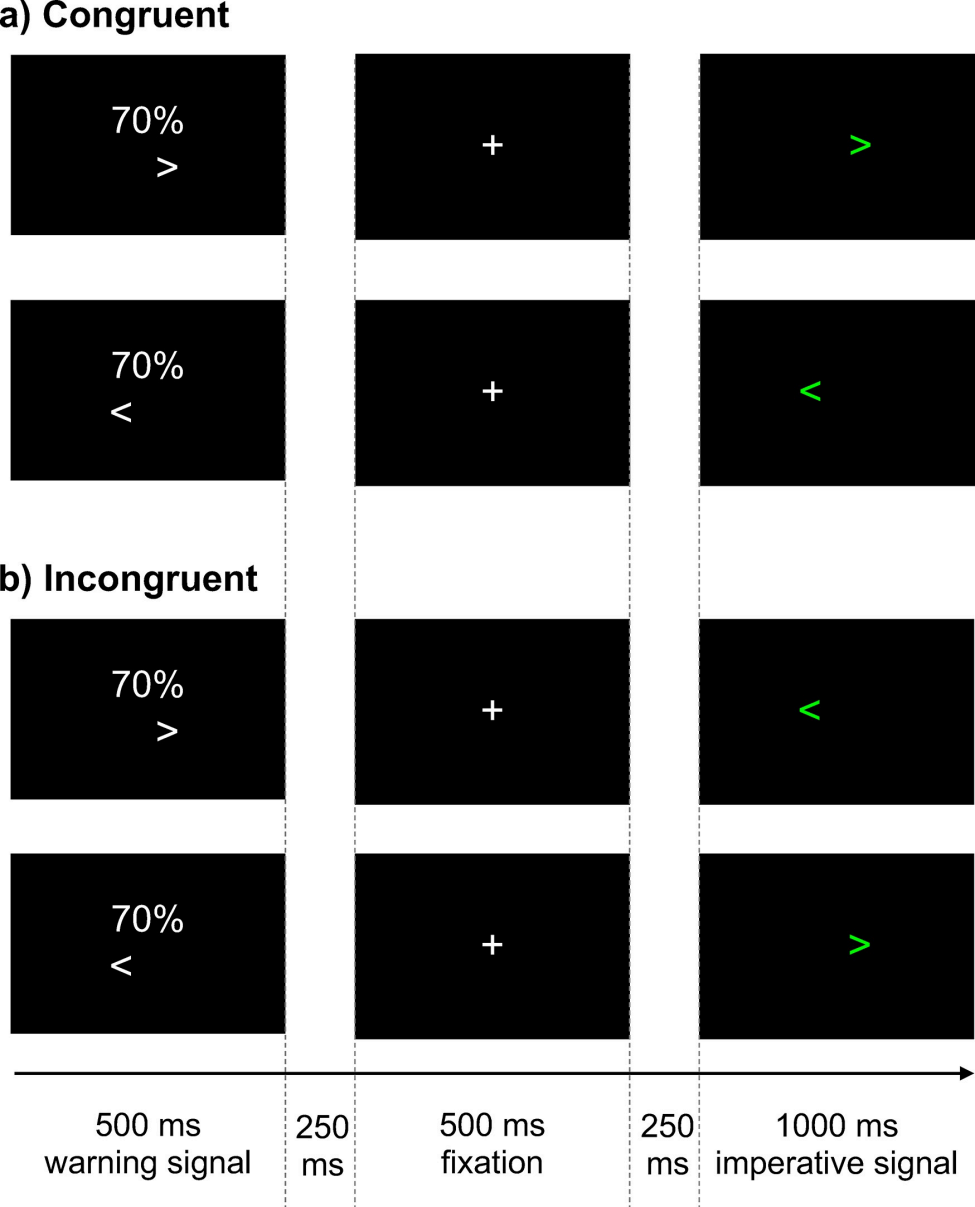
Experimental trials. On a computer monitor, participants were presented a 500 ms warning signal which indicated a 70% probability of the upcoming imperative signal pointing either to the right (>) or left (<). Following the warning signal, a blank screen (250 ms), a fixation cross (500 ms), and another blank screen (250 ms) were presented. Following these, a 1000 ms green coloured imperative signal (> or <) was presented either in the same (**a**: congruent trials) or opposite (**b**: incongruent trials) direction to the warning signal. Participants were required to respond with a button consistent with the direction of the arrow in the imperative signal.

However, one may argue that the different priming of left vs. right responses, and more broadly decision-based processes as well, may be influenced by other mechanisms besides prior probability. Specifically, the provision of a warning signal (‘prime’), regardless of its validity, prior to the imperative signal (‘target’) might invoke *response priming* with the visual similarity between the warning and imperative signal possibly invoking *perceptual priming* (see Fig 2 and ‘Experimental procedure’ section). Response priming in the current study was tested with the presence of ‘catch’ trials (i.e., trials where the warning signal was *not* followed by an imperative signal) such that a negligible influence of response priming would be reflected by participants correctly withholding their responses on catch trials (i.e., lower false alarm rate). Perceptual priming was tested by ensuring that the warning signal prime and the imperative signal target utilized the same symbol (arrows as per Fig 2), with any perceptual priming effects biasing sensory evidence and being reflected as superior model fits of consistent evidence-accumulation parameters (*v* parameter in Fig 1).

Secondly, our model assumes that observed responses are based on a stimulus-driven decision-making process rather than a “fast guess” process in which stimulus onset triggers a guessed response that is chosen prior to stimulus onset [3, 33, 34]. Fast guesses are known to occur on a subset of trials in bias manipulations of prior probability and payoffs, with the fast guess response usually favouring the more probable and higher payoff response [3, 33]. However, given that this is especially the case in *extreme* levels of bias where it is optimal to allow stimulus onset to trigger the fast guess response [3, 35], the current study utilised a moderate level of bias to minimize fast guesses. Specifically, as illustrated in Fig 2, the warning signal indicated, prior to stimulus onset, a 70% bias towards a particular response. A second purpose of including catch trials was such that if participants utilised a fast-guessing strategy, one would expect a higher false alarm rate (and vice versa). Furthermore, an exponentially-weighted moving average RT filter was utilised to determine, for each participant separately, the cut-off point below which RTs were reflective of a fast-guessing strategy (i.e., 50% accuracy in responses). Based on these procedures and the evidence (see ‘Data processing’ section), we are confident in our assumption that filtered responses are reflective of a stimulus-driven decision-making process.

In summary, using a moderately biased choice RT task and the racing diffusion model, the current study investigated the effect of prior probability, in the different biasing procedures, on response execution across the lifespan. We investigated whether: 1) response thresholds are lower and/or 2) mean accumulation rate higher and/or 3) response execution time shorter for the accumulator corresponding to the higher probability stimulus. We further investigated whether these effects were more prominent under the block-wise than trial-wise biasing procedure, and in older compared to young adults.

## Materials and methods

### Participants

Twenty-one healthy young adults (mean age = 26.76 years; SD = 5.85 years; range = 19 to 40 years; all but two self-declaring right-handed dominance) and 20 healthy older adults (mean age = 67.95 years; SD = 5.12 years; range = 61 to 84 years; all but one self-declaring right-handed dominance) were recruited between 2017 and 2018 from the local university and the community. The Mini-Mental State Examination [36] was used to screen older adults for cognitive integrity and everyone scored within the normal range (score ≥ 26; [37]). Prior to participating in the study, all participants provided written informed consent. De-identified data were used for all analyses with re-identification only possible through a secure master coding file so that individual data could be withdrawn prior to publication if requested by a participant. The study was approved by the Tasmanian Human Research Ethics Committee Network and conducted in accordance with the Declaration of Helsinki.

### Experimental procedure

On separate days, at least 24 h apart, participants attended two sessions (1.5–2.5 h duration). One session only involved the behavioural task, and the other session involved the behavioural task as well as non-invasive brain stimulation which has been published previously [32]. Given the known influence of brain stimulation on RT [38–40], data from the session only involving the behavioural task is analysed here.

Participants undertook a choice RT task in which button press responses to simple visual stimuli, using their left or right index fingers, were required. The likelihood of a particular response was conveyed using a warning stimulus presented prior to the imperative stimulus. Thus, on each trial, a warning signal, “70% >” or “70% <”, was first presented for 500 ms indicating the true probability of the upcoming imperative signal pointing in the same direction. Following this, a blank screen (250 ms), a central fixation cross (500 ms), and another blank screen (250 ms) were presented in the listed temporal order. Following these, a 1000 ms green imperative signal, pointing either to the right, “>”, or to the left, “<”, was presented on a computer monitor. Participants were instructed to respond, as quickly and accurately as possible, using their right (to “>”) or left (to “<”) index finger by pressing buttons on a USB response pad (The Black Box ToolKit, Sheffield, UK). Thus, trials were classified as either *congruent* trials (70% of trials)–where the warning and imperative signals pointed in the same direction–or *incongruent* trials (30% of trials)–where the warning and imperative signals pointed in opposite directions, as depicted in Fig 2. Participants were provided with feedback 750 ms after every trial, composing of either a) the RT (in seconds) if a correct response was made, or b) “Incorrect press” if an incorrect response was made, or c) “Missed” if no response was made. Consequently, the total trial duration was 3250 ms and the inter-trial interval was set to 750 ms. PsychoPy2 v1.84.2 was used to present all experimental stimuli [41].

Twenty blocks of 65 trials each were presented using *block-wise* and *trial-wise* bias procedures to investigate differences in bias control. Specifically, for block-wise biasing, the warning signal remained the same across the whole block of trials and hence the more likely response option across the whole block of trials remained the same. For trial-wise biasing, the warning signal changed pseudorandomly from trial-to-trial in a block and consequently the more likely response option changed pseudorandomly from trial-to-trial in a block, with an equal number of left- and right-handed response options in a block of trials. Overall, participants completed 10 blocks each of the block-wise and trial-wise bias, counterbalanced across participants. Five trials in each block were ‘catch’ trials (i.e., no imperative signal followed the warning signal) where participants were required to withhold their response. These catch trials were implemented to ensure that participants were not anticipating the direction and timing of the imperative signal [32, 42]. Of the remaining 60 trials, 42 were congruent trials and 18 were incongruent trials (considering that the warning signal was always accurate and always indicated a 70% probability).

### Data processing

For one participant two blocks of data could not be collected due to technical difficulties. Furthermore, prior to model fitting, catch trials (on average, the false alarm rate was low, with participants correctly withholding their response on 94.9% of catch trials suggesting negligible response priming effects and that participants did not utilize a wholly anticipatory ‘fast guessing’ strategy) and trials on which no response was made (average of 1.5% across all participants) were excluded.

In addition to utilising catch trials and ensuring that the prior probability bias manipulation was not too extreme, the inclusion of any fast guess trials in the final sample was further minimized by applying two types of RT filters. Firstly, RTs less than 200 ms were discarded. Secondly, for each participant and each bias type, RTs less than ‘cut-off’ RTs–found using an exponentially-weighted moving average (EWMA) of accuracy after sorting by RT (starting point = 0.5; weighting alpha parameter = 0.1; cut-off accuracy threshold = 0.6)–were discarded [43, 44]. Overall, 1265 of 48,306 trials were removed (2.6%) based on these two procedures. Of the 1265 trials, 819 trials were classified as having RTs below 200 ms, 151 trials were classified as having RTs below the cut-off determined by the EWMA procedure, and 295 trials were classified as meeting both the aforementioned criteria. Consequently, due to these various procedures, histograms of RTs where the imperative signal was congruent with the biasing warning signal (i.e., the condition where fast guesses would be most apparent) showed no evidence of participants utilising a fast-guessing strategy (see S3 Fig).

### Racing diffusion model

#### Model fitting

The racing diffusion model utilised in the current study, as illustrated in Fig 1, includes several parameters for each of the two accumulators (one for a left and one for a right response). Mean evidence-accumulation rate, *v*, was parameterised separately for the (correct) accumulator that matched the stimulus (‘matching *v*’) and the (incorrect) accumulator that mismatched the stimulus (‘mismatching *v*’). In addition, to incorporate bias effects, *v* was parameterised as a ‘bias-right’ accumulation rate parameter, *v*_br_, by scaling to the minimum accumulation rate and adding it to the right accumulator (and subtracting from the left accumulator). Thus, *v*_br_ would range between +1, indicating maximum right bias, and -1, indicating maximum left bias, with 0 indicating no bias. For example, if the rates for left and right accumulator are *v*_left_ and *v*_right_ before the addition of bias, and denoting the minimum of these two values as *d*_v_ (typically the rate for the mismatching accumulator), the rates after the addition of bias (with subscripts “right,bias” and “left,bias”) become:

$$v_{right,bias}=v_{right}+d_{v}*v_{br}$$ (a)

$$v_{left,bias}=v_{left}-d_{v}*v_{br}$$ (b)

Note that scaling *v*_br_ by *d*_v_ ensures that the rates remain non-negative.

Threshold and nondecision time were parameterised as *b* and *t*_0_, respectively, for every bias direction (left and right) and accumulator (left and right) combination. Given that accumulators raced with a positive evidence-accumulation rate towards a positive threshold, RTs are described by a shifted-Wald distribution, with the shift equal to the nondecision time component [29, 30]. Of note, i) the start-point noise parameter, *A*, was kept constant across all conditions, and ii) threshold results are reported in terms of *B* = *b–A* ≥ 0, enforcing the constraint *b* ≥ *A* (i.e., accumulation cannot begin above the threshold) and since *A* was constant across all conditions, differences in *B* reflect pure threshold effects.

#### Sampling

Hierarchical Bayesian techniques, using an MCMC algorithm as implemented in the *Dynamic Models of Choice* R functions [45], were utilized to obtain the entire posterior probability distribution of the parameters. First, for each participant separately, posterior distributions were estimated using multiple chains (precisely, three times the number of parameters). Diffuse and independent normal priors were set for all parameters. Specifically, means and standard deviations for *B*, *matching v*, *mismatching v*, *v*_br_, and *t*_0_ were set to 2, 3, 1, 0, 0, and 2, 4, 4, 1, and 0.5, respectively. A lower bound of 0 was used for all parameters except for *v*_br_ (set to -1) with no upper bound except for *v*_br_ and *t*_0_ having an upper bound of +1 and 1000 ms, respectively.

Individual fits starting from points sampled from the priors provided start-points for hierarchical models, which assumed independent normal population distributions, and were fit separately for each age group (young and older) and bias procedure (block-wise and trial-wise). Hyper-priors for population mean parameters were assumed to be the same independent normal priors as for individual fits. Hyper-priors for population standard deviations were exponential with a mean of 1 for all parameters. Each MCMC chain during hierarchical fitting was thinned by 10 (i.e., only one iteration in every 10 was retained) and a 5% probability of migration used until all chains had similar posterior likelihoods. Hierarchical sampling continued until Gelman’s multivariate potential scale reduction factor was less than 1.1 [46], indicating mixing, stationarity, and convergence. Lastly, a final set of 250 iterations per chain (after thinning) was run, with the resulting samples used in further analyses. Convergence was confirmed by visual inspection of trace plots of the final samples.

#### Model selection

For each age group and bias type combination, candidate hierarchical models were constructed where bias (i.e., warning signal biasing a left- or right-hand response) affected one or more of the threshold, evidence-accumulation rate, and nondecision time parameters, resulting in seven different models. Without a bias effect there are two threshold parameters (one for the left accumulator and one for the right accumulator), two rate parameters (one for the accumulator matching the stimulus and one for the mismatching accumulator) and two nondecision time parameters (one for the left accumulator and one for the right). In each case this number doubles with an added bias effect with different values estimated depending on whether the bias is to the left or right. We constructed acronyms for each model from the bias-affected parameters. In three models, each with 8 parameters, the bias affects: i) only threshold (*B* model), ii) only accumulation rate (*v* model), iii) only nondecision time (*t*_0_ model). In three more models, each with 10 parameters, bias affects: iv) threshold and accumulation rate (*Bv* model), v) threshold and nondecision time (*Bt*_0_ model), vi) accumulation rate and nondecision time (*vt*_0_ model). In the seventh model, with 12 parameters, bias affects vii) threshold, accumulation rate, and nondecision time (*Bvt*_0_ model). The 7 hierarchical models were fit for each age group and bias type combination, resulting in a grand total of 28 hierarchical models. Model selection was conducted using the *deviance information criterion* (DIC), which penalizes goodness of fit measured by likelihood with a complexity penalty [47], where smaller values indicate a preferred model. As with most such information criteria, a difference of 10 units or more indicates a substantial preference [45].

Converging evidence on the relative importance of each parameter type in explaining the effect of bias was evaluated in several ways. First, we compared DIC values over the models with only one bias effect, with the best (lowest DIC) model indicating its parameter type is most influential. Second, we compared models with two bias effects, with the parameter type being omitted in the best model indicated as least influential. Finally, for the overall best (i.e., the model with the lowest DIC value among all seven) and second-best models, the relative importance of each parameter type in simulating bias effects on two key behavioural measures of bias, response probability and correct RT, were evaluated by removing the bias effect on each parameter type while keeping it on the remaining parameter types [48]. For rate bias *v*_br_ was set to 0 for each posterior parameter sample. For *B* and *t*_0_ the four parameter estimates were reduced to two for each posteior sample by averaging over the bias manipulation. Random samples from the transformed posteriors were then used to generate 100 posterior predictive data sets, each consisting of a RT and choice for each trial, condition, and participant. The resulting distribution over 100 data sets of differences between incongruent and congruent conditions captures the model’s average prediction and uncertianty about the effects averaged over trials, conditions, and participants of parametrer types that retained bias effects. We report the median and 95% credible interval of the bias effect predicted by the reduced model divided by the effect predicted by the full model. When this ratio is close to 1, the removed parameter effect has little impact on the bias effect, whereas ratios less than 1 indicate that the removed parameter has a substantial role in explaining the predicted bias. Occasionaly the ratio can be greater than 1, indicating a trade-off where the removed parameter masks a larger bias caused by the remaining parameter effect(s).

## Results

The data and analyses for the current study are available at https://osf.io/yh2rp/.

### Model selection

In the trial-wise bias type, for both young and older adults, the model where bias varied over *B*, *v*, and *t*_0_ (*Bvt*_0_ model) had the smallest DIC whereas in the block-wise bias type the model where bias varied over *B* and *t*_0_ (*Bt*_0_ model) had the smallest DIC (Table 1). These two models were clearly better than the others in all cases. For scenarios where bias was allowed to vary over two parameters, the *Bt*_0_ model had the smallest DIC for all age group and bias type combinations, indicating that accumulation rate had the least role in explaining bias effects. For scenarios where bias was only allowed to vary over a single parameter, the threshold only, *B*, model had the smallest DIC for all combinations, indicating it is the most influential parameter type.

**Table 1:** Model fit.

	Young adults Block-wise bias	Young adults Trial-wise bias	Older adults Block-wise bias	Older adults Trial-wise bias
***v***	708	601	823	694
***t***_**0**_	299	211	228	163
***B***	217	121	135	140
***vt***_**0**_	114	92	207	99
***Bv***	182	122	123	44
***Bt***_**0**_	0 (-38798)	5	0 (-28270)	38
***Bvt***_**0**_	6	0 (-38708)	9	0 (-28525)

Model fit measures (values represent differences in DIC relative to the model with the smallest DIC, indicated with a 0 and the DIC value in parentheses) for models where bias was allowed to vary over unique combinations of threshold (B), accumulation rate (v), and nondecision time (t_0_) for all age group (young and older adults) and bias type (block-wise and trial-wise bias) combinations, separately.

Although the mean *B* and *t*_0_ parameter values for the two best overall models were in the predicted directions (i.e., lower *B* and *t*_0_ values for congruent compared to the incongruent parameters; S1 Table), there was a lack of consistency for *Bvt*_0_ model’s accumulation rate estimates, with 5 out of 8 values causing a *negative* bias effect (i.e., better performance in the incongruent condition, see S2 Table), suggesting overfitting by the *Bvt*_0_ model and inconsistent perceptual priming effects.

This interpretation is supported by posterior predictive analysis of the *Bvt*_0_ model. As shown in Table 2, the predicted correct RT bias effect (i.e., incongruent correct RT–congruent correct RT) and the predicted accuracy bias effect (i.e., incongruent proportion correct–congruent proportion correct) were very close to the observed effects. With no threshold bias effect there was substantial misfit relative to the full model, with anywhere from half to all of the biasing in accuracy and correct RT removed. Omitting the nondecision time bias effect had a more moderate effect causing a reduction of about one third in correct RT bias (nondecision time has no effect on accuracy). In contrast, omitting the accumulation rate bias effect led to inconsistent results, sometimes causing either relatively small increases or decreases in the predicted bias effects. These results suggest that bias effects were primarily driven by threshold and secondarily by nondecision time. Posterior predictive analysis of the *Bt*_0_ model (Table 3) supports this conclusion. The quality of predictions for both accuracy and correct RT bias effects is comparable to the *Bvt*_0_ model.

**Table 2:** Posterior predictive analysis for Bvt_0_ model.

			Omitted effect				
			*v*		*B*		*t*_0_
	RT	ACC	RT	ACC	RT	ACC	RT
**YB**	-3 [–5, –1]	0.01 [0.001, 0.03]	0.87 [0.75, 1.01]	0.76 [0.49, 1.03]	0.47 [0.30, 0.65]	0.47 [0.20, 0.83]	0.66 [0.54, 0.79]
**YT**	-2 [–5, 1]	0.01 [0.004, 0.02]	0.99 [0.84, 1.14]	0.95 [0.62, 1.42]	0.34 [0.12, 0.59]	0.21 [-0.07, 0.58]	0.67 [0.51, 0.85]
**OB**	1 [–2, 5]	0.01 [0.002, 0.02]	1.00 [0.90, 1.13]	0.97 [0.76, 1.31]	0.31 [0.16, 0.42]	0.13 [-0.06, 0.28]	0.69 [0.54, 0.86]
**OT**	1 [–3, 5]	0.01 [-0.001, 0.02]	1.17 [1.04, 1.31]	1.41 [1.07, 2.05]	0.19 [-0.02, 0.38]	-0.03 [-0.27, 0.25]	0.61 [0.46, 0.77]

For each age group (Y: young adults; O: older adults) and bias type (B: block-wise bias; T: trial-wise bias) combination, differences between the Bvt_0_ model predicted bias effect and observed bias effect (response time in milliseconds as RT and proportion correct for accuracy as ACC) as well as the ratios of the bias effect with threshold, B, accumulation rate, v, or nondecision time, t_0_, omitted are reported for both, correct response-time (RT) and accuracy (ACC). Note, numbers within square brackets represent the lower and upper bounds of the 95% credible intervals and for t_0_ only RT is presented.

**Table 3:** Posterior predictive analysis for Bt_0_ model.

			Omitted effect		
			*B*		*t*_0_
	RT	ACC	RT	ACC	RT
**YB**	-3 [–5, –1]	0.01 [0.00, 0.02]	0.22 [0.09, 0.34]	0.14 [-0.01, 0.31]	0.74 [0.62, 0.89]
**YT**	-2 [–5, 0]	0.01 [0.00, 0.02]	0.32 [0.18, 0.49]	0.13 [-0.07, 0.31]	0.66 [0.51, 0.85]
**OB**	2 [–3, 6]	0.01 [0.00, 0.02]	0.31 [0.19, 0.43]	0.14 [-0.02, 0.34]	0.68 [0.54, 0.85]
**OT**	2 [–1, 6]	0.01 [0.00, 0.02]	0.56 [0.42, 0.71]	0.46 [0.23, 0.70]	0.43 [0.29, 0.61]

For each age group (Y: young adults; O: older adults) and bias type (B: block-wise bias; T: trial-wise bias) combination, differences between the Bt_0_ model predicted bias effect and observed bias effect (response time in milliseconds as RT and proportion correct for accuracy as ACC) as well as the ratios of the bias effect with threshold, B, or nondecision time, t_0_, omitted are reported for both, correct response-time (RT) and accuracy (ACC). Note, numbers within square brackets represent the lower and upper bounds of the 95% credible intervals and for t_0_ only RT is presented.

**Fig 3:**
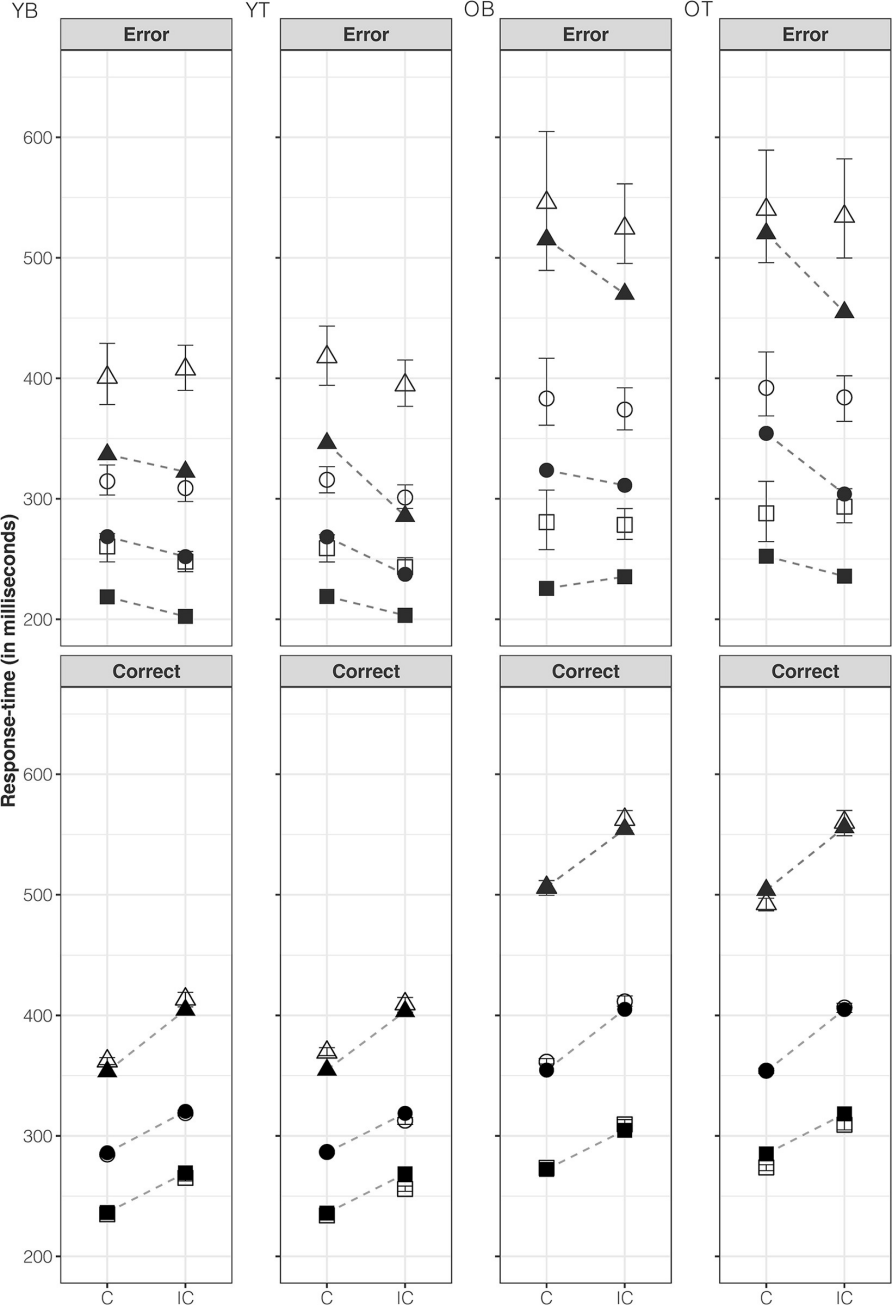
Response time. Observed (filled) and *Bt*_0_ model-predicted (unfilled) 10^th^ (square), 50^th^ (circle), and 90^th^ (triangle) error (top panel) and correct (bottom panel) response time percentiles plotted, in milliseconds, separately for each age group–young (Y) and older (O) adults–as well as each bias type–block-wise (B) and trial-wise (T). Observed data points between congruent (C) and incongruent (IC) trial types are connected via dashed lines. Error bars for model-predicted response times represent 95% credible intervals.

**Fig 4:**
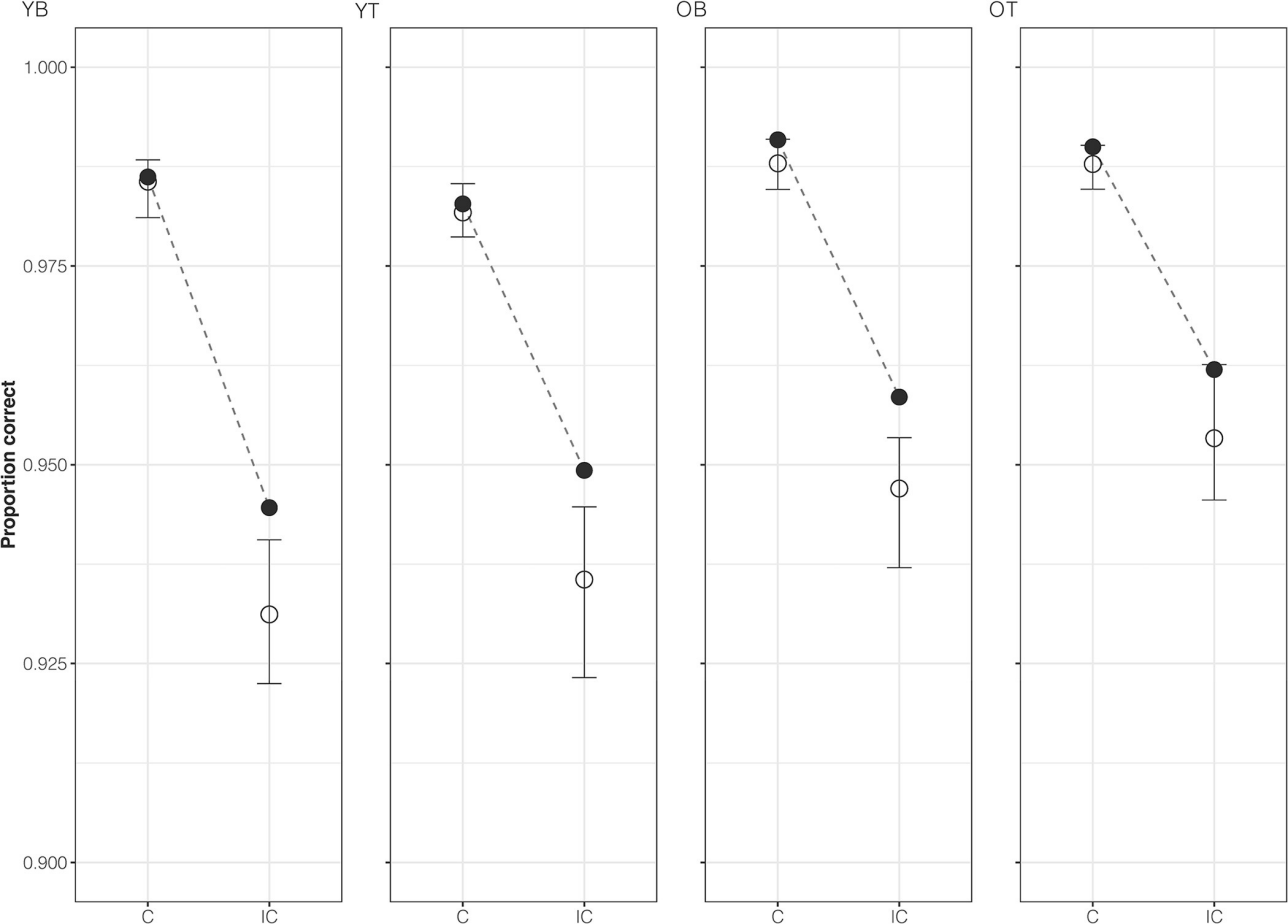
Accuracy. Observed (filled) and *Bt*_0_ model-predicted (unfilled) proportion of correct responses plotted separately for each age group–young (A) and older (B) adults–as well as each bias type–block-wise (1) and trial-wise (2). Observed data points between congruent (C) and incongruent (IC) trial types are connected via dashed lines. Error bars for model-predicted proportion of correct responses represent 95% credible intervals.

Considering all the above converging evidence, the *Bt*_0_ model provides the best account of all conditions. Observed distributions of RT, for both correct and error responses, and accuracy, along with predictions for the *Bt*_0_ model, are illustrated in Figs 3 and 4, respectively. Overall, as expected, older adults had slower but more accurate responses than young adults. RTs were slower on correct incongruent than correct congruent responses, whereas the opposite pattern was observed for error responses. The model captures these trends and provides a good quantitative account, with the observed data falling within the model-predicted 95% credible intervals. However, some over-estimation occurred for error RTs along with a small (~1%) under-estimation of the proportion of correct incongruent responses. This misfit was not addressed by the *Bvt*_0_ model (see S1 and S2 Figs).

### Parameter tests

Parameter tests were conducted on the *Bt*_0_ model for thresholds, *B*, and nondecision time, *t*_0_, parameters separately. Table 4 presents the median estimates for *B* and *t*_0_ at every combination of all experimental factors with Table 5 presenting the *differences* in median estimates (differences considered *credibly significant* if the 95% CI did not contain 0).

**Table 4:** Median parameter estimates.

	Young adults				Older adults			
	Block-wise		Trial-wise		Block-wise		Trial-wise	
	IC	C	IC	C	IC	C	IC	C
***B***	1.18 [1.14, 1.23]	0.95 [0.93, 0.98]	1.04 [1.01, 1.08]	0.88 [0.86, 0.91]	1.20 [1.16, 1.24]	1.01 [0.98, 1.04]	1.23 [1.19, 1.27]	1.07 [1.04, 1.11]
***t***_**0**_	176 [172, 180]	167 [165, 169]	184 [181, 188]	175 [173, 177]	216 [211, 221]	201 [198, 204]	218 [212, 223]	188 [185, 192]

Median estimates for threshold (B) and nondecision time (t_0_, in milliseconds) parameters at every combination of age group (Young and Older adults), bias type (Trial-wise and block-wise), and trial type (Congruent and Incongruent) experimental factors. Values within square brackets represent the lower and upper bounds of the 95% credible intervals.

**Table 5:** Differences in median parameter estimates.

		Bias effect		Response caution	Average speed of nondecision processes (ms)
		*B*	*t*_0_ (ms)		
**Young**	*Block-wise*	**0.23 [0.19, 0.27]**	**9 [4, 13]**	-	-
	*Trial-wise*	**0.16 [0.13, 0.19]**	**9 [5, 13]**		
	*Block-wise vs*. *trial-wise*	**0.07 [0.02, 0.12]**	-0.2 [–6, 5]	**0.11 [0.07, 0.15]**	**-8 [–12, –5]**
**Older**	*Block-wise*	**0.19 [0.15, 0.23]**	**15 [9, 21]**	-	-
	*Trial-wise*	**0.15 [0.11, 0.20]**	**29 [23, 35]**		
	*Block-wise vs*. *trial-wise*	0.04 [-0.02, 0.10]	**-14 [–23, –6]**	**-0.044 [-0.087, -0.003]**	**6 [1, 10]**
**Young vs. Older**	*Block-wise*	0.04 [-0.02, 0.09]	-7 [–14, 1]	-0.039 [-0.080, 0.003]	**-37 [–41, –33]**
	*Trial-wise*	0.01 [-0.05, 0.06]	**-20 [–27, –13]**	**-0.19 [-0.23, -0.15]**	**-23 [–27, –19]**
	*Block-wise vs*. *trial-wise*	0.03 [-0.05, 0.11]	**14 [3, 24]**	**0.15 [0.09, 0.21]**	**-14 [–20, –8]**

Differences in median estimates to infer a) bias effects (incongruent–congruent) for threshold (B) and nondecision time (t_0_, in milliseconds) parameters, b) response caution (averaged incongruent and congruent threshold parameters), and c) average speed of nondecision processes (averaged incongruent and congruent nondecision time parameters). Differences in block-wise, trial-wise, and between block-wise and trial-wise bias types are reported for young, older, and between young and older adults with certain differences, for b) and c), not theoretically possible (‘-’). For block- and trial-wise bias types, positive values indicate a larger incongruent, than congruent, value, whereas for block-wise vs. trial-wise bias type and young vs. older adult comparisons, positive values indicate a greater bias effect of the former. Note, values within square brackets represent the lower and upper bounds of the 95% credible intervals and bolded values indicate differences being credibly different from 0.

For *B*, in both young and older adults, credibly significant bias effects were observed in block- and trial-wise bias types with incongruent thresholds being greater than congruent thresholds. However, only for young adults was the threshold bias effect credibly greater in the block- than trial-wise bias type. Focussing on response *caution* (i.e., average of incongruent and congruent *B* parameters), comparing young and older adults, though caution was greater for older adults in both bias types, it was only credibly significant in the trial-wise bias type with this difference being credibly greater than in the block-wise bias type.

For *t*_0_, in both young and older adults, credibly significant bias effects were observed in block- and trial-wise bias types with incongruent nondecision time being greater than congruent nondecision time. However, only for older adults was the nondecision time bias effect credibly greater in the trial- than block-wise bias type (14 ms [6–23]). Furthermore, comparing young and older adults, though the nondecision time bias effect was greater for older adults in both bias types, it was only credibly significant in the trial-wise bias type (20 ms [13–27]) with this difference being credibly greater than in the block-wise bias type (14 ms [3–24]). Focussing on the average speed of nondecision processing (i.e., average of incongruent and congruent *t*_0_ parameters), comparing young and older adults, average nondecision processing speed was credibly greater for older adults in both bias types with this difference being credibly greater in the block- than trial-wise bias type.

In summary, older adults, compared to young adults, exhibited a greater nondecision time bias effect in trial- than block-wise bias type. In addition, older adults, compared to young adults, exhibited greater response caution in the trial- than block-wise bias type but exhibited longer average nondecision processing in the block- than trial-wise bias type.

## Discussion

The current study provides novel insights, from the racing diffusion model perspective, into the selective nature of prior probability effects. Specifically, besides the canonical effect of prior probability on response thresholds, a clear influence was also observed on a nondecision-based process of response execution, but not on accumulation rates. These observations were apparent across the lifespan (i.e., in both healthy young and older adults) as well as across both (i.e., block- and trial-wise) biasing procedures.

In line with previous research in perceptual decision-making [2–9, 26] and recognition memory [8, 10, 11], a strong effect of prior probability on response thresholds was observed in the current study. This was not only based on complexity penalized goodness of fit, which consistently favoured bias varying over the *B* parameter, but also in the posterior predictive analysis where omitting the threshold bias effect resulted in significant and consistent misfit. In both young and older adults, a significant biasing effect (i.e., incongruent threshold greater than congruent threshold) was observed in the block- and trial-wise biasing procedures, speaking to the robustness of the effect. Considering response caution (i.e., averaged incongruent and congruent parameter), older adults were more cautious than young adults, especially in the trial-wise biasing procedure. Given the well-established age-associated decline in cognitive processes such as processing speed and executive functioning [49], it is unsurprising that older adults were more cautious in the more labile and cognitively demanding trial-wise biasing condition.

However, the effect of prior probability was not entirely selective. That is, besides the canonical and well-reported effect on response thresholds, a significant effect was also observed on the nondecision-based process of response execution. In the current study, prior probability had a significant effect on response execution as evidenced by the best model always allowing bias to vary over the nondecision time parameter as well as the posterior predictive analysis exhibiting moderate misfit when omitting a bias effect on nondecision time. This robust effect was observed in both age groups and biasing conditions as the incongruent response execution time was greater (slower) than congruent response execution time. Recent research suggests an important role of GABAergic inhibitory pathways between the primary motor cortices in putatively subserving the observed response execution biasing effect [32]. Specifically, in the time period between the warning signal delivering prior probability information and the imperative signal, greater cortical inhibition was observed in the hand that was biased *away* from the cue (i.e., incongruent condition) than the hand that was biased towards the cue (i.e., congruent condition). Speculatively, this greater cortical inhibition is likely to have an influence by requiring more motoneurons and/or greater firing rates to generate a response, resulting in the observed increase in response execution time during the incongruent condition. Furthermore, the slower incongruent response execution time was most apparent in older adults in the trial-wise biasing condition. In addition to the aforementioned greater demands of the trial-wise bias condition and cognitive declines associated with healthy ageing, known age-related motor deficits such as movement slowing are likely to have played a role [27, 28].

Lastly, in the current study, we did not obtain convincing evidence for the influence of prior probability on the rate of evidence-accumulation. Though in the more challenging trial-wise biasing condition, and to a greater extent in older adults, some evidence of a rate effect was observed. The nature of this effect was small and inconsistent, most often exhibited in the opposite direction to that expected with a higher rate for the *less* likely outcome. In contrast, previous reports of prior probability effects on the quality of sensory evidence found that the more likely outcome has a higher rate of evidence-accumulation and the less likely outcome a lower rate [9, 12]. A potential explanation of this discrepancy is that these studies not only required more difficult perceptual discriminations than that required in our study but also utilised a different evidence-accumulation model (drift diffusion model vs. the racing diffusion model utilized here).

Future studies may build upon insights gained from the current research in numerous ways, some of which are outlined here. First, the generality of the observed non-selective effect of prior probability could be tested by investigating: i) other evidence-accumulation models such as the drift-diffusion model [2], ii) a range of prior probabilities (vs. only the 70% utilized here), iii) other bias manipulations such as payoffs that often have similar canonical effects as prior probability [6], and iv) more difficult (error prone) choices. Second, computational approaches that jointly model the variation in behavioural *and* neurophysiological data could provide further insights into the neural mechanisms involved [50]. Lastly, given the differences observed here due to the healthy ageing process, the question of how these effects vary in clinical groups exhibiting cognitive and motor deficits is of importance.

In conclusion, the current study investigated, in groups of healthy young and older adults across different bias conditions, the selective influence assumption of prior probability using the racing diffusion model. Besides the expected influence of prior probability on response thresholds, a clear although smaller effect was also observed on the nondecision-based process of response execution time in both age groups and both bias conditions.

## Supporting information

S1 Table Median parameter estimates for the Bt_0_ model. Parameter values for both accumulators (LEFT: left response; RIGHT: right response) in the model where bias (‘toleft’ and ‘toright’ indicating leftward and rightward bias, respectively) was allowed to vary over unique combinations of threshold (B) and nondecision time (t_0_ in seconds) for all age group (young and older adults) and bias type (block-wise and trial-wise bias) combinations, separately. In addition, the accumulator that matches and mismatches the stimulus is termed as ‘v.true’ and ‘v.false’, respectively.
(DOCX)

S2 Table Median parameter estimates for the Bvt_0_ model. Parameter values for both accumulators (LEFT: left response; RIGHT: right response) in the model where bias (‘toleft’ and ‘toright’ indicating leftward and rightward bias, respectively) was allowed to vary over unique combinations of threshold (B), accumulation rate (v), and nondecision time (t_0_ in seconds) for all age group (young and older adults) and bias type (block-wise and trial-wise bias) combinations, separately. In addition, the accumulator that matches and mismatches the stimulus is termed as ‘v.true’ and ‘v.false’, respectively.
(DOCX)

S1 Fig Response time. Observed (filled) and *Bvt*_0_ model-predicted (unfilled) 10^th^ (square), 50^th^ (circle), and 90^th^ (triangle) error (top panel) and correct (bottom panel) response-time percentiles plotted, in milliseconds, separately for each age group–young (Y) and older (O) adults–as well as each bias type–block-wise (B) and trial-wise (T). Observed data points between congruent (C) and incongruent (IC) trial types are connected via dashed lines. Error bars for model-predicted response-times represent 95% credible intervals.
(DOCX)

S2 Fig Accuracy. Observed (filled) and *Bvt*_0_ model-predicted (unfilled) proportion of correct responses plotted separately for each age group–young (A) and older (B) adults–as well as each bias type–block-wise (1) and trial-wise (2). Observed data points between congruent (C) and incongruent (IC) trial types are connected via dashed lines. Error bars for model-predicted proportion of correct responses represent 95% credible intervals.
(DOCX)

S3 Fig Participant-level congruent response-time histograms. For each participant, response times during the congruent (imperative signal congruent with warning signal) condition are plotted as a histogram (between 0 and 1000 ms with a 25 ms bin width, noting that response times below 200 ms were discarded).
(DOCX)

## References

[1] Gold JI, Shadlen MN. Neural computations that underlie decisions about sensory stimuli. Trends Cogn Sci. 2001 Jan 1;5(1):10–6. doi: 10.1016/s1364-6613(00)01567-9 11164731

[2] Ratcliff R, McKoon G. The diffusion decision model: Theory and data for two-choice decision tasks [Internet]. Vol. 20, Neural Computation. MIT Press; 2008 [cited 2021 May 17]. p. 873–922. Available from: http://direct.mit.edu/neco/article-pdf/20/4/873/817277/neco.2008.12-06-420.pdf 18085991 10.1162/neco.2008.12-06-420 PMC2474742

[3] Simen P, Contreras D, Buck C, Hu P, Holmes P, Cohen JD. Reward Rate Optimization in Two-Alternative Decision Making: Empirical Tests of Theoretical Predictions. J Exp Psychol Hum Percept Perform [Internet]. 2009 Dec [cited 2021 Nov 2];35(6):1865–97. Available from: /record/2009-22869-017?doi=1 doi: 10.1037/a0016926 19968441 PMC2791916

[4] Forstmann BU, Brown S, Dutilh G, Neumann J, Wagenmakers EJ. The neural substrate of prior information in perceptual decision making: a model-based analysis. Front Hum Neurosci. 2010;0:40. doi: 10.3389/fnhum.2010.00040 20577592 PMC2889713

[5] Leite FP, Ratcliff R. What cognitive processes drive response biases? A diffusion model analysis. Vol. 6, Judgment and Decision Making. 2011.

[6] Mulder MJ, Wagenmakers EJ, Ratcliff R, Boekel W, Forstmann BU. Bias in the Brain: A Diffusion Model Analysis of Prior Probability and Potential Payoff. J Neurosci [Internet]. 2012 Feb 15 [cited 2021 Jul 20];32(7):2335–43. Available from: https://www.jneurosci.org/content/32/7/2335 doi: 10.1523/JNEUROSCI.4156-11.2012 22396408 PMC6621823

[7] van Ravenzwaaij D, Dutilh G, Wagenmakers EJ. A diffusion model decomposition of the effects of alcohol on perceptual decision making. Psychopharmacol 2011 2194 [Internet]. 2011 Aug 13 [cited 2021 Nov 2];219(4):1017–25. Available from: https://link.springer.com/article/10.1007/s00213-011-2435-9 21842158 10.1007/s00213-011-2435-9 PMC3266508

[8] White CN, Poldrack RA. Decomposing bias in different types of simple decisions. J Exp Psychol Learn Mem Cogn. 2014;40(2):385–98. doi: 10.1037/a0034851 24245536

[9] Dunovan K, Wheeler ME. Computational and neural signatures of pre and post-sensory expectation bias in inferior temporal cortex. Sci Reports 2018 81 [Internet]. 2018 Sep 5 [cited 2021 Nov 2];8(1):1–13. Available from: https://www.nature.com/articles/s41598-018-31678-x doi: 10.1038/s41598-018-31678-x 30185928 PMC6125426

[10] Criss AH. Differentiation and Response Bias in Episodic Memory: Evidence From Reaction Time Distributions. J Exp Psychol Learn Mem Cogn [Internet]. 2010 Mar [cited 2021 Nov 2];36(2):484–99. Available from: /record/2010-03582-015?doi=1 doi: 10.1037/a0018435 20192544

[11] Arnold NR, Bröder A, Bayen UJ. Empirical validation of the diffusion model for recognition memory and a comparison of parameter-estimation methods. Psychol Res 2014 795 [Internet]. 2014 Oct 4 [cited 2021 Nov 2];79(5):882–98. Available from: https://link.springer.com/article/10.1007/s00426-014-0608-y 25281426 10.1007/s00426-014-0608-y PMC4534506

[12] Hanks TD, Mazurek ME, Kiani R, Hopp E, Shadlen MN. Elapsed Decision Time Affects the Weighting of Prior Probability in a Perceptual Decision Task. J Neurosci [Internet]. 2011 Apr 27 [cited 2021 Jul 20];31(17):6339–52. Available from: https://www.jneurosci.org/content/31/17/6339 doi: 10.1523/JNEUROSCI.5613-10.2011 21525274 PMC3356114

[13] Wagenmakers EJ, Ratcliff R, Gomez P, McKoon G. A diffusion model account of criterion shifts in the lexical decision task. J Mem Lang. 2008 Jan 1;58(1):140–59. doi: 10.1016/j.jml.2007.04.006 19122740 PMC2330283

[14] Bogacz R, Wagenmakers EJ, Forstmann BU, Nieuwenhuis S. The neural basis of the speed-accuracy tradeoff. Trends in Neurosciences. 2010. doi: 10.1016/j.tins.2009.09.002 19819033

[15] Donkin C, Little DR, Houpt JW. Assessing the speed-accuracy trade-off effect on the capacity of information processing. J Exp Psychol Hum Percept Perform [Internet]. 2014 [cited 2021 Nov 2];40(3):1183–202. Available from: /record/2014-09038-001 doi: 10.1037/a0035947 24635202

[16] Rae B, Heathcote A, Donkin C, Averell L, Brown S. The hare and the tortoise: Emphasizing speed can change the evidence used to make decisions. J Exp Psychol Learn Mem Cogn [Internet]. 2014 [cited 2021 Nov 2];40(5):1226–43. Available from: /record/2014-16300-001 doi: 10.1037/a0036801 24797438

[17] van Maanen L, Forstmann BU, Keuken MC, Wagenmakers EJ, Heathcote A. The impact of MRI scanner environment on perceptual decision-making. Behav Res Methods 2015 481 [Internet]. 2015 Feb 21 [cited 2021 Nov 2];48(1):184–200. Available from: https://link.springer.com/article/10.3758/s13428-015-0563-6 10.3758/s13428-015-0563-6 PMC4819926 25701105

[18] Weigard A, Beltz A, Reddy SN, Wilson SJ. Characterizing the role of the pre-SMA in the control of speed/accuracy trade-off with directed functional connectivity mapping and multiple solution reduction. Hum Brain Mapp [Internet]. 2019 Apr 15 [cited 2021 Nov 2];40(6):1829–43. Available from: https://onlinelibrary.wiley.com/doi/full/10.1002/hbm.24493 30569619 10.1002/hbm.24493 PMC6865688

[19] Palada H, Searston RA, Persson A, Ballard T, Thompson MB. An evidence accumulation model of perceptual discrimination with naturalistic stimuli. J Exp Psychol Appl [Internet]. 2020 [cited 2021 Nov 2];26(4):671–91. Available from: /record/2020-31142-001 doi: 10.1037/xap0000272 32378930

[20] Sewell DK, Stallman A. Modeling the effect of speed emphasis in probabilistic category learning. Comput Brain Behav. 2019 Oct 29;3(2):129–52.

[21] Rafiei F, Rahnev D. Qualitative speed-accuracy tradeoff effects that cannot be explained by the diffusion model under the selective influence assumption. Sci Reports 2021 111 [Internet]. 2021 Jan 8 [cited 2021 Nov 2];11(1):1–19. Available from: https://www.nature.com/articles/s41598-020-79765-2 doi: 10.1038/s41598-020-79765-2 33420181 PMC7794484

[22] Evans NJ. Think fast! The implications of emphasizing urgency in decision-making. Cognition. 2021 Sep 1;214:104704. doi: 10.1016/j.cognition.2021.104704 33975126

[23] Voss A, Rothermund K, Voss J. Interpreting the parameters of the diffusion model: An empirical validation. Mem Cogn 2004 327 [Internet]. 2004 [cited 2021 Nov 2];32(7):1206–20. Available from: https://link.springer.com/article/10.3758/BF03196893 10.3758/bf03196893 15813501

[24] Lerche V, Voss A. Experimental validation of the diffusion model based on a slow response time paradigm. Psychol Res 2017 836 [Internet]. 2017 Dec 9 [cited 2021 Nov 2];83(6):1194–209. Available from: https://link.springer.com/article/10.1007/s00426-017-0945-8 29224184 10.1007/s00426-017-0945-8

[25] Dambacher M, Hübner R. Time pressure affects the efficiency of perceptual processing in decisions under conflict. Psychol Res 2014 791 [Internet]. 2014 Feb 2 [cited 2021 Nov 2];79(1):83–94. Available from: https://link.springer.com/article/10.1007/s00426-014-0542-z 24487728 10.1007/s00426-014-0542-z

[26] Garton R, Reynolds A, Hinder MR, Heathcote A. Equally flexible and optimal response bias in older compared to younger adults. Psychol Aging [Internet]. 2019 Sep 1 [cited 2021 May 17];34(6):821–35. Available from: /record/2019-15318-001?doi=1 doi: 10.1037/pag0000339 30907604

[27] Seidler RD, Bernard JA, Burutolu TB, Fling BW, Gordon MT, Gwin JT, et al. Motor control and aging: Links to age- related brain structural, functional, and biochemical effects. Neurosci Biobehav Rev. 2010;34(5):721–33. doi: 10.1016/j.neubiorev.2009.10.005 19850077 PMC2838968

[28] Dully J, McGovern DP, O’Connell RG. The impact of natural aging on computational and neural indices of perceptual decision making: A review. Vol. 355, Behavioural Brain Research. Elsevier B.V.; 2018. p. 48–55. 29432793 10.1016/j.bbr.2018.02.001

[29] Leite FP, Ratcliff R. Modeling reaction time and accuracy of multiple-alternative decisions. Attention, Perception, Psychophys 2010 721 [Internet]. 2010 [cited 2021 Jul 20];72(1):246–73. Available from: https://link.springer.com/article/10.3758/APP.72.1.246 20045893 10.3758/APP.72.1.246 PMC2805113

[30] Tillman G, Zandt T Van, Logan GD. Sequential sampling models without random between-trial variability: the racing diffusion model of speeded decision making. Psychon Bull Rev 2020 275 [Internet]. 2020 May 18 [cited 2021 Jul 20];27(5):911–36. Available from: https://link.springer.com/article/10.3758/s13423-020-01719-6 32424622 10.3758/s13423-020-01719-6

[31] Bestmann S, Harrison LM, Blankenburg F, Mars RB, Haggard P, Friston KJ, et al. Influence of Uncertainty and Surprise on Human Corticospinal Excitability during Preparation for Action. Curr Biol [Internet]. 2008 [cited 2017 Jul 31];18(10):775–80. Available from: http://ac.els-cdn.com/S096098220800537X/1-s2.0-S096098220800537X-main.pdf?_tid=ec601c34-7586-11e7-8ae4-00000aacb35e&acdnat=1501460981_936c2acc9d8c938e18f108fc2865e854 doi: 10.1016/j.cub.2008.04.051 18485711 PMC2387198

[32] Puri R, Hinder MR. Response bias reveals the role of interhemispheric inhibitory networks in movement preparation and execution. Neuropsychologia [Internet]. 2022 Jan;165:108120. Available from: https://linkinghub.elsevier.com/retrieve/pii/S0028393221003730 doi: 10.1016/j.neuropsychologia.2021.108120 34915037

[33] Noorbaloochi S, Sharon D, McClelland JL. Payoff Information Biases a Fast Guess Process in Perceptual Decision Making under Deadline Pressure: Evidence from Behavior, Evoked Potentials, and Quantitative Model Comparison. J Neurosci [Internet]. 2015 Aug 5 [cited 2021 Jul 20];35(31):10989–1011. Available from: https://www.jneurosci.org/content/35/31/10989 doi: 10.1523/JNEUROSCI.0017-15.2015 26245962 PMC6605280

[34] Ratcliff R. Theoretical Interpretations of the Speed and Accuracy of Positive and Negative Responses. Psychol Rev [Internet]. 1985 Apr [cited 2022 Aug 31];92(2):212–25. Available from: /record/1985-19264-001 3991839

[35] Bogacz R, Brown E, Moehlis J, Holmes P, Cohen JD. The physics of optimal decision making: A formal analysis of models of performance in two-alternative forced-choice tasks. Psychol Rev. 2006 Oct;113(4):700–65. doi: 10.1037/0033-295X.113.4.700 17014301

[36] Dick JP, Guiloff RJ, Stewart A, Blackstock J, Bielawska C, Paul EA, et al. Mini-mental state examination in neurological patients. J Neurol Neurosurg Psychiatry [Internet]. 1984;47(5):496–9. Available from: http://jnnp.bmj.com/content/47/5/496.short doi: 10.1136/jnnp.47.5.496 6736981 PMC1027826

[37] Jacqmin-Gadda H, Fabrigoule C, Commenges D, Dartigues JF. A 5-year longitudinal study of the Mini-Mental State Examination in normal aging. Am J Epidemiol [Internet]. 1997;145(6):498–506. Available from: http://www.ncbi.nlm.nih.gov/pubmed/9063339 doi: 10.1093/oxfordjournals.aje.a009137 9063339

[38] Romaiguère P, Possamaï C a, Hasbroucq T. Motor cortex involvement during choice reaction time: a transcranial magnetic stimulation study in man. Brain Res [Internet]. 1997;755(2):181–92. Available from: http://www.ncbi.nlm.nih.gov/pubmed/9175886 doi: 10.1016/s0006-8993(97)00095-4 9175886

[39] Burle B, Bonnet M, Vidal F, Possamaï CA, Hasbroucq T. A transcranial magnetic stimulation study of information processing in the motor cortex: Relationship between the silent period and the reaction time delay. Psychophysiology [Internet]. 2002 Mar 1 [cited 2021 Nov 2];39(2):207–17. Available from: https://onlinelibrary.wiley.com/doi/full/10.1111/1469-8986.3920207 12212670 10.1017/S0048577202010077

[40] Hasbroucq T, Kaneko H, Akamatsu M, Possamaï CA. The time-course of preparatory spinal and cortico-spinal inhibition: An H-reflex and transcranial magnetic stimulation study in man. Exp Brain Res. 1999;124(1):33–41. doi: 10.1007/s002210050597 9928787

[41] Peirce J, Gray JR, Simpson S, MacAskill M, Höchenberger R, Sogo H, et al. PsychoPy2: Experiments in behavior made easy. Behav Res Methods [Internet]. 2019 Feb 15 [cited 2021 May 17];51(1):195–203. Available from: 10.3758/s13428-018-01193-y 30734206 PMC6420413

[42] Hinder MR, Puri R, Kemp S, Waitzer S, Reissig P, Stöckel T, et al. Distinct modulation of interhemispheric inhibitory mechanisms during movement preparation reveals the influence of cognition on action control. Cortex. 2018;99(October):13–29. doi: 10.1016/j.cortex.2017.10.002 29121485

[43] Vandekerckhove J, Tuerlinckx F. Diffusion model analysis with MATLAB: A DMAT primer. Behav Res Methods [Internet]. 2008 Feb [cited 2021 May 17];40(1):61–72. Available from: https://link.springer.com/article/10.3758/BRM.40.1.61 18411528 10.3758/brm.40.1.61

[44] Nunez MD, Vandekerckhove J, Srinivasan R. How attention influences perceptual decision making: Single-trial EEG correlates of drift-diffusion model parameters. J Math Psychol. 2017 Feb 1;76:117–30. doi: 10.1016/j.jmp.2016.03.003 28435173 PMC5397902

[45] Heathcote A, Lin YS, Reynolds A, Strickland L, Gretton M, Matzke D. Dynamic models of choice. Behav Res Methods 2018 512 [Internet]. 2018 Jun 29 [cited 2021 Nov 2];51(2):961–85. Available from: https://link.springer.com/article/10.3758/s13428-018-1067-y 10.3758/s13428-018-1067-y 29959755

[46] Gelman A, Rubin DB. Inference from iterative simulation using multiple sequences. Stat Sci [Internet]. 1992 Nov 1 [cited 2021 May 17];7(4):457–72. Available from: https://projecteuclid.org/journals/statistical-science/volume-7/issue-4/Inference-from-Iterative-Simulation-Using-Multiple-Sequences/10.1214/ss/1177011136.full

[47] Spiegelhalter DJ, Best NG, Carlin BP, Van Der Linde A. Bayesian measures of model complexity and fit. J R Stat Soc Ser B (Statistical Methodol [Internet]. 2002 Oct 1 [cited 2021 Jun 6];64(4):583–639. Available from: https://onlinelibrary.wiley.com/doi/full/10.1111/1467-9868.00353

[48] Strickland L, Loft S, Remington RW, Heathcote A. Racing to remember: A theory of decision control in event-based prospective memory. Psychol Rev [Internet]. 2018 Nov 1 [cited 2021 Nov 2];125(6):851–87. Available from: /record/2018-37935-001 doi: 10.1037/rev0000113 30080068

[49] Deary IJ, Corley J, Gow AJ, Harris SE, Houlihan LM, Marioni RE, et al. Age-associated cognitive decline. Br Med Bull [Internet]. 2009 Dec 1 [cited 2018 May 2];92(1):135–52. Available from: http://www.ncbi.nlm.nih.gov/pubmed/19776035 doi: 10.1093/bmb/ldp033 19776035

[50] Turner BM, Forstmann BU, Love BC, Palmeri TJ, Van Maanen L. Approaches to analysis in model-based cognitive neuroscience. J Math Psychol [Internet]. 2017 [cited 2017 Mar 14];76:65–79. Available from: www.elsevier.com/locate/jmp doi: 10.1016/j.jmp.2016.01.001 31745373 PMC6863443

