# Supplementary material for: What mechanisms mediate prior probability effects on rapid-choice decision-making?
Source: PLoS One. 2023 Jul 7;18(7):e0288085. doi: 10.1371/journal.pone.0288085 (PMC10328325; doi:10.1371/journal.pone.0288085)
Supplement: S1 Table — Parameter values for both accumulators (LEFT: left response; RIGHT: right response) in the model where bias (‘toleft’ and ‘toright’ indicating leftward and rightward bias, respectively) was allowed to vary over unique combinations of threshold (B) and nondecision time (t0 in seconds) for all age group (young and older adults) and bias type (block-wise and trial-wise bias) combinations, separately. In addition, the accumulator that matches and mismatches the stimulus is termed as ‘v.true’ and ‘v.false’, respectively. (DOCX) [file pone.0288085.s001.docx]

**S1 Table***.* Parameter values for both accumulators (LEFT: left response; RIGHT: right response) in the model where bias (‘toleft’ and ‘toright’ indicating leftward and rightward bias, respectively) was allowed to vary over unique combinations of threshold (B) and nondecision time (t_0_ in seconds) for all age group (young and older adults) and bias type (block-wise and trial-wise bias) combinations, separately. In addition, the accumulator that matches and mismatches the stimulus is termed as ‘v.true’ and ‘v.false’, respectively.

|  | Young adults (block-wise bias) | Young adults (trial-wise bias) | Older adults (block-wise bias) | Older adults (trial-wise bias) |
| --- | --- | --- | --- | --- |
| B.toleft.LEFT | 0.950 | 0.929 | 1.062 | 1.123 |
| B.toright.LEFT | 1.292 | 1.016 | 1.163 | 1.240 |
| B.toleft.RIGHT | 1.070 | 1.067 | 1.239 | 1.214 |
| B.toright.RIGHT | 0.957 | 0.833 | 0.960 | 1.026 |
| t0.toleft.LEFT | 0.169 | 0.172 | 0.196 | 0.187 |
| t0.toright.LEFT | 0.163 | 0.190 | 0.229 | 0.226 |
| t0.toleft.RIGHT | 0.189 | 0.178 | 0.203 | 0.209 |
| t0.toright.RIGHT | 0.166 | 0.179 | 0.205 | 0.189 |
| v.true | 7.597 | 7.427 | 5.745 | 5.994 |
| v.false | 0.864 | 0.711 | 0.047 | 0.204 |
