# Supplementary material for: What mechanisms mediate prior probability effects on rapid-choice decision-making?
Source: PLoS One. 2023 Jul 7;18(7):e0288085. doi: 10.1371/journal.pone.0288085 (PMC10328325; doi:10.1371/journal.pone.0288085)
Supplement: S1 Fig — Observed (filled) and Bvt0 model-predicted (unfilled) 10th (square), 50th (circle), and 90th (triangle) error (top panel) and correct (bottom panel) response-time percentiles plotted, in milliseconds, separately for each age group–young (Y) and older (O) adults–as well as each bias type–block-wise (B) and trial-wise (T). Observed data points between congruent (C) and incongruent (IC) trial types are connected via dashed lines. Error bars for model-predicted response-times represent 95% credible intervals. (DOCX) [file pone.0288085.s003.docx]

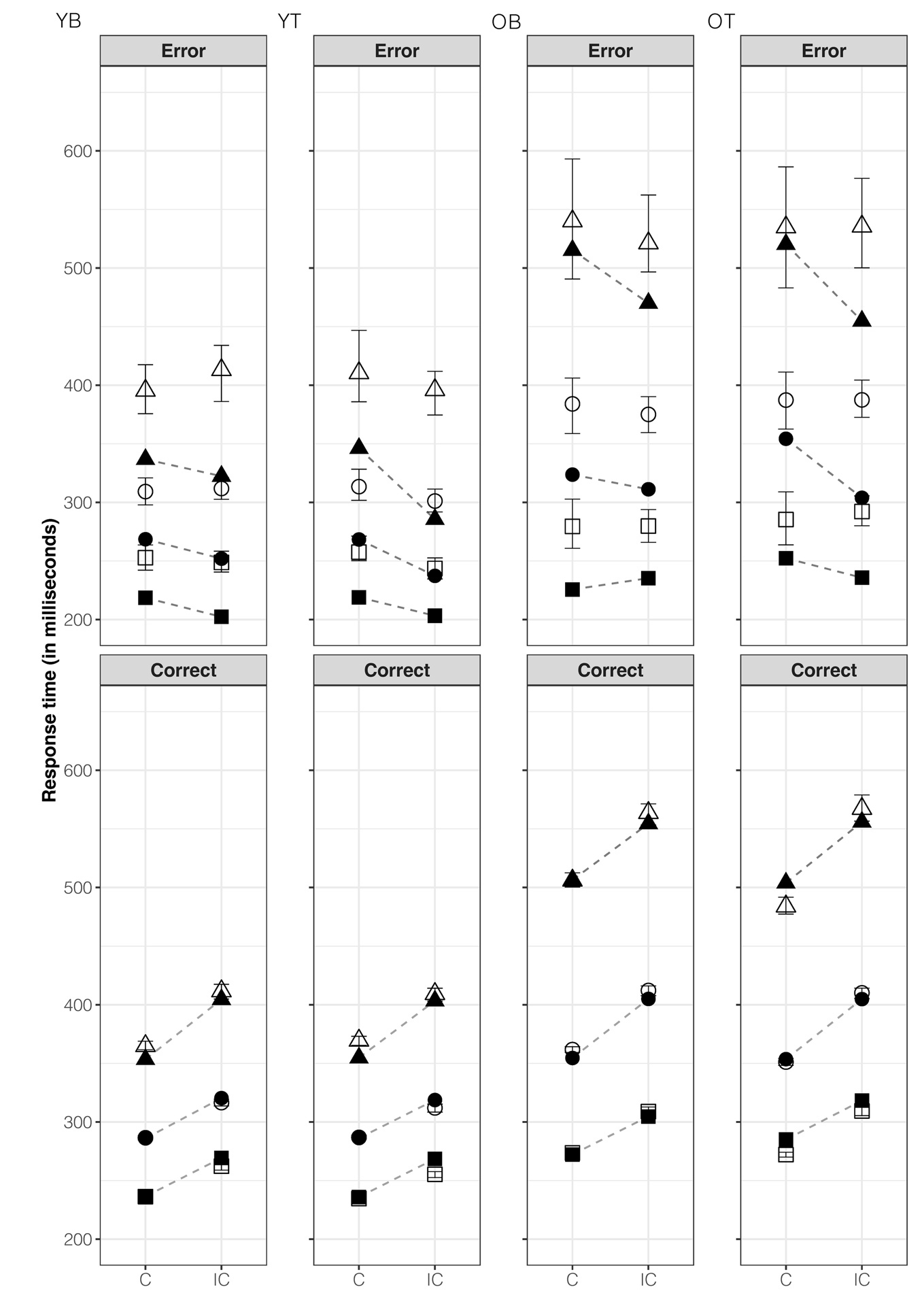


**S3 Fig.** **Response time**. Observed (filled) and *Bvt*_0_ model-predicted (unfilled) 10^th^ (square), 50^th^ (circle), and 90^th^ (triangle) error (top panel) and correct (bottom panel) response-time percentiles plotted, in milliseconds, separately for each age group – young (Y) and older (O) adults – as well as each bias type – block-wise (B) and trial-wise (T). Observed data points between congruent (C) and incongruent (IC) trial types are connected via dashed lines. Error bars for model-predicted response-times represent 95% credible intervals.
