# Supplementary material for: What mechanisms mediate prior probability effects on rapid-choice decision-making?
Source: PLoS One. 2023 Jul 7;18(7):e0288085. doi: 10.1371/journal.pone.0288085 (PMC10328325; doi:10.1371/journal.pone.0288085)
Supplement: S2 Fig — Observed (filled) and Bvt0 model-predicted (unfilled) proportion of correct responses plotted separately for each age group–young (A) and older (B) adults–as well as each bias type–block-wise (1) and trial-wise (2). Observed data points between congruent (C) and incongruent (IC) trial types are connected via dashed lines. Error bars for model-predicted proportion of correct responses represent 95% credible intervals. (DOCX) [file pone.0288085.s004.docx]

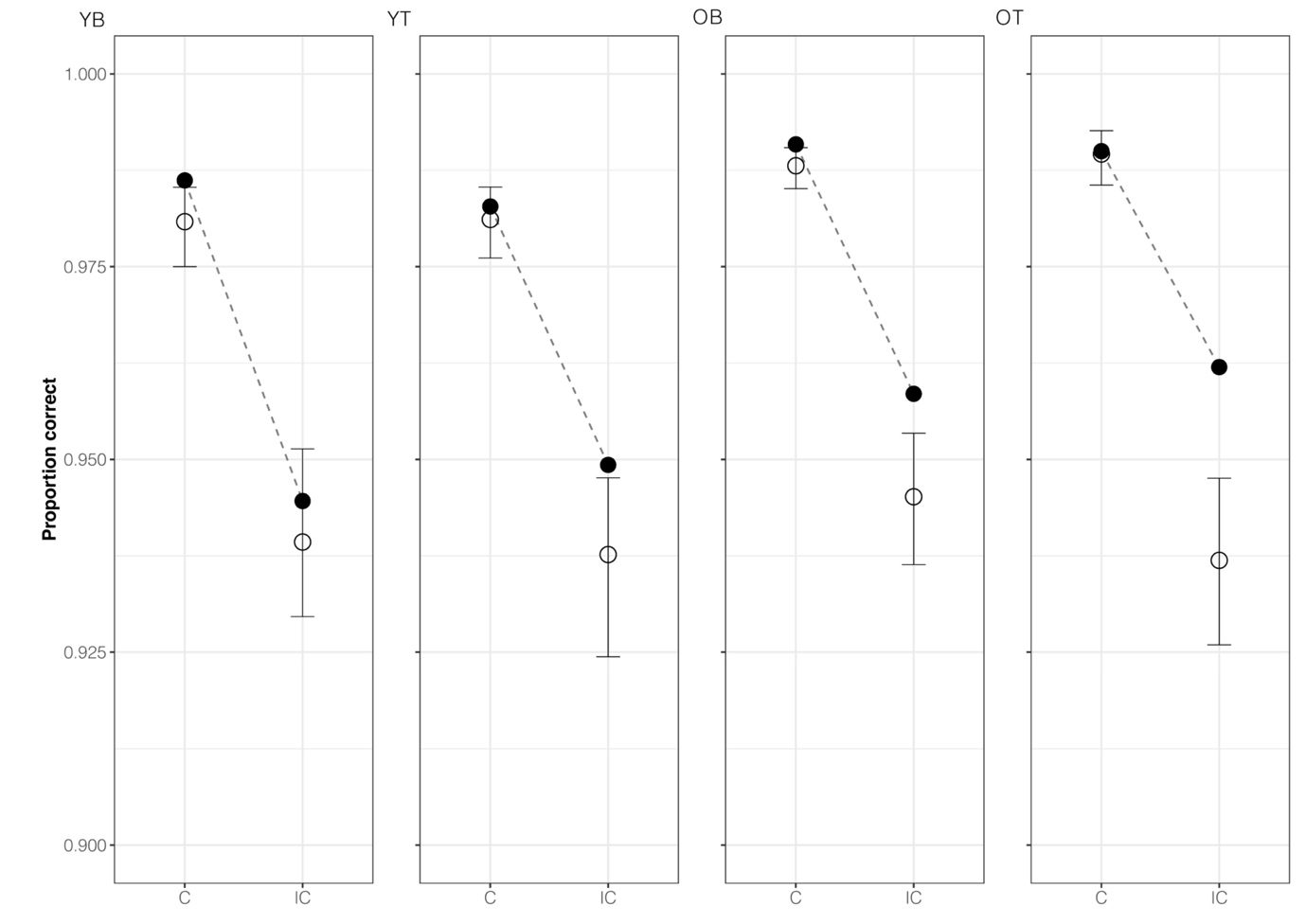


**S4 Fig. Accuracy**. Observed (filled) and *Bvt*_0_ model-predicted (unfilled) proportion of correct responses plotted separately for each age group – young (A) and older (B) adults – as well as each bias type – block-wise (1) and trial-wise (2). Observed data points between congruent (C) and incongruent (IC) trial types are connected via dashed lines. Error bars for model-predicted proportion of correct responses represent 95% credible intervals.
