# Supplementary material for: What mechanisms mediate prior probability effects on rapid-choice decision-making?
Source: PLoS One. 2023 Jul 7;18(7):e0288085. doi: 10.1371/journal.pone.0288085 (PMC10328325; doi:10.1371/journal.pone.0288085)
Supplement: S3 Fig — For each participant, response times during the congruent (imperative signal congruent with warning signal) condition are plotted as a histogram (between 0 and 1000 ms with a 25 ms bin width, noting that response times below 200 ms were discarded). (DOCX) [file pone.0288085.s005.docx]

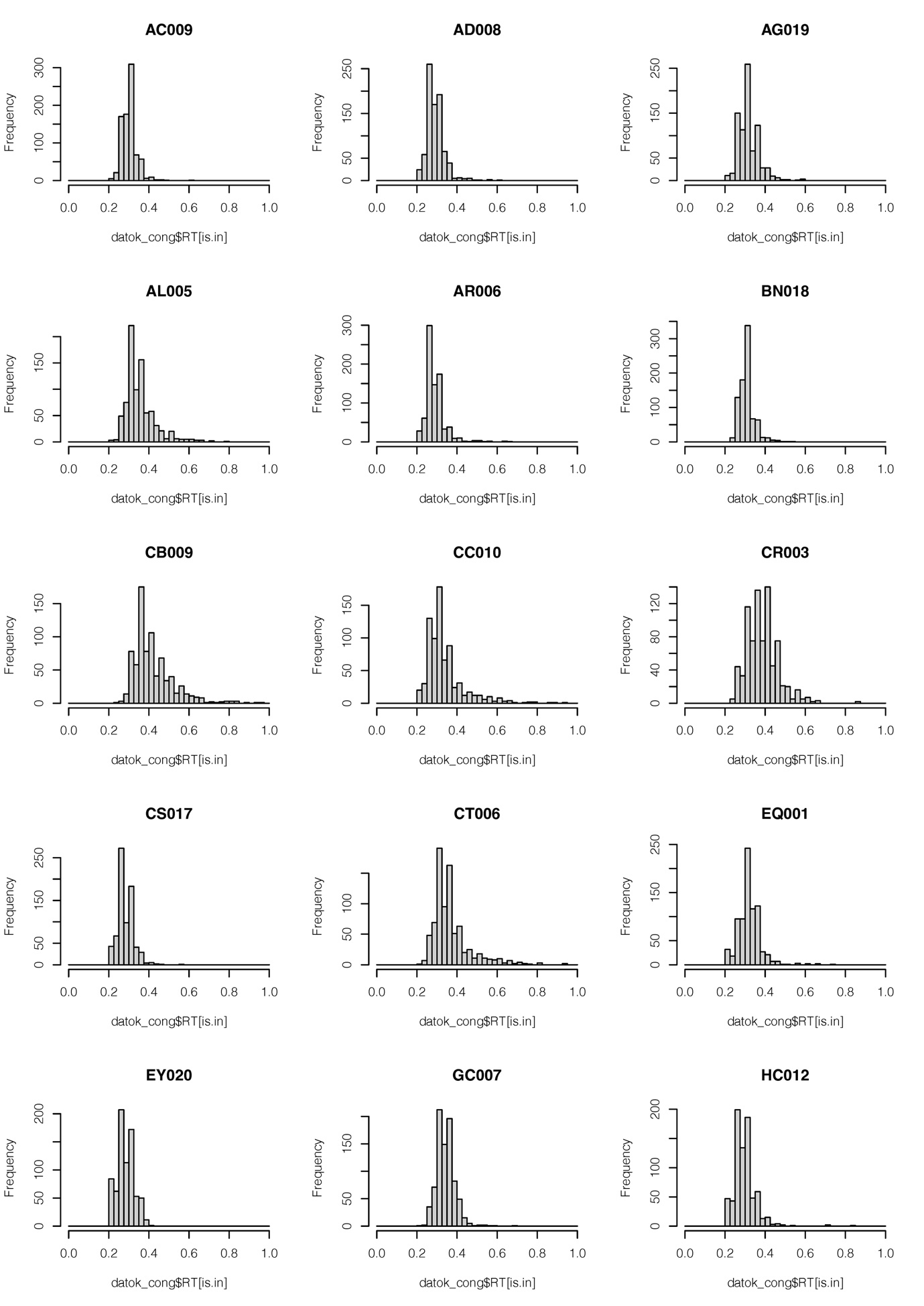


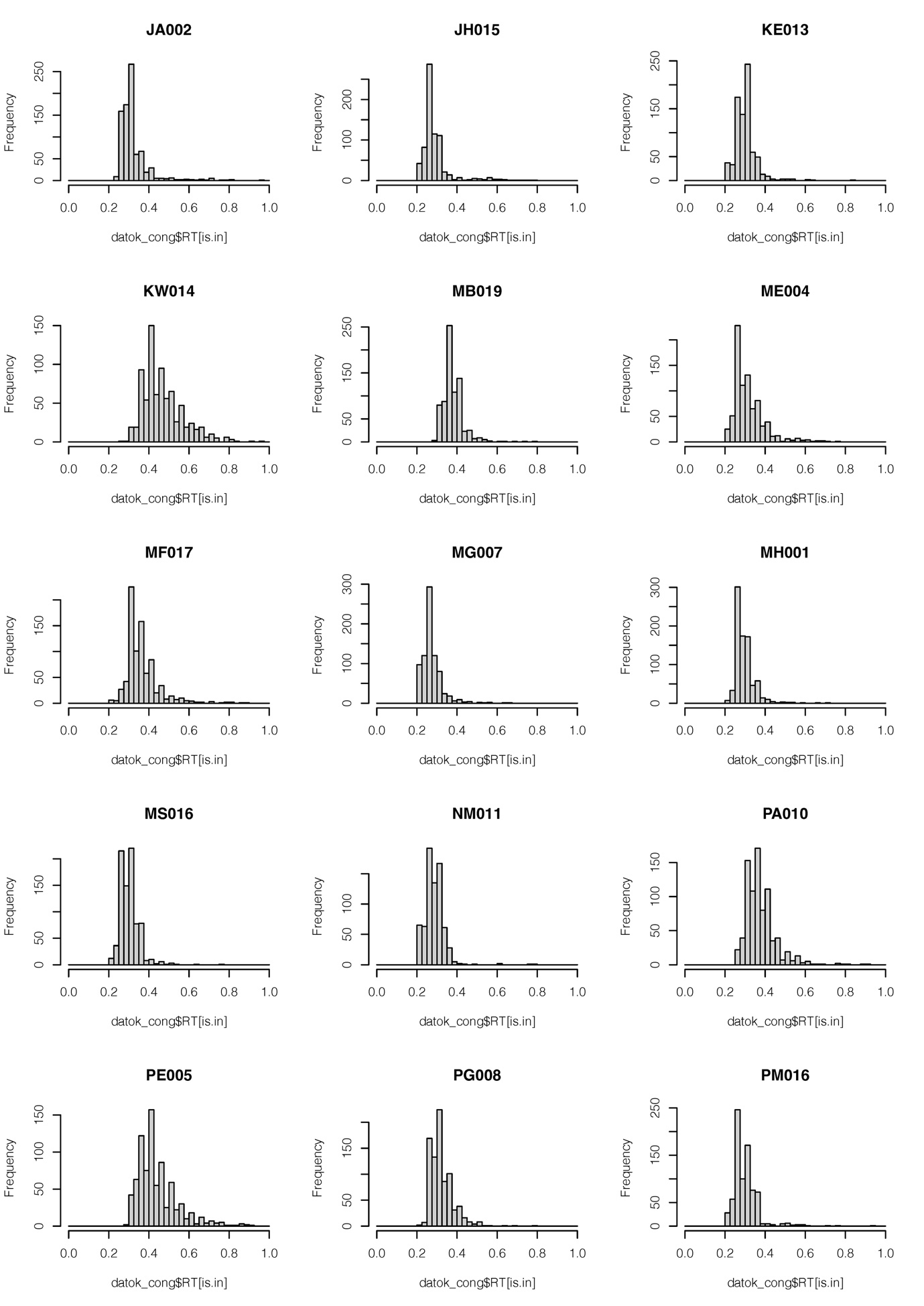


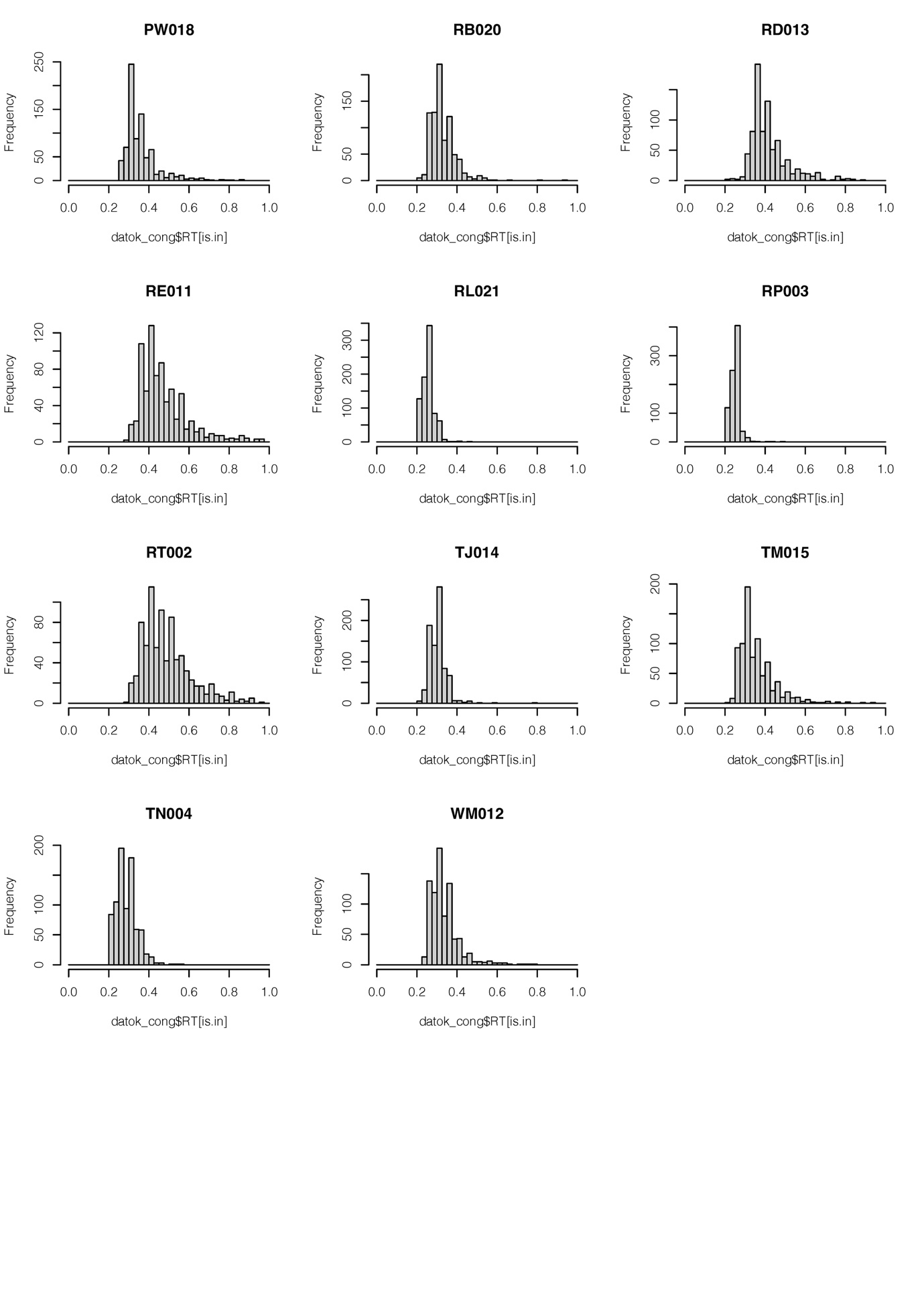


**S5 Fig. Participant-level congruent response-time histograms**. For each participant, RTs during the congruent (imperative signal congruent with warning signal) condition are plotted as a histogram (between 0 and 1000 ms with a 25 ms bin width, noting that RTs below 200 ms were discarded).
