# Supplementary material for: What mechanisms mediate prior probability effects on rapid-choice decision-making?
Source: PLoS One. 2023 Jul 7;18(7):e0288085. doi: 10.1371/journal.pone.0288085 (PMC10328325; doi:10.1371/journal.pone.0288085)
Supplement: Attachment — Submitted filename: PlosOneMarch28Review.pdf [file pone.0288085.s006.pdf]

The study investigates the effect of prior probability on response thresholds using a racing diffusion model to account for response time and accuracy. They also investigated the effect of parameters on healthy younger versus healthy older adults. They found that the prior probability has a significant effect on response thresholds and clear influence on response execution across lifespan.

The introduction was clear with well explained reasoning. The Methods and Results explained in detail and Discussion In general, the sentences could be a bit shorter. That would make the reading easier. I have some minor comments:

Introduction:

- Page 5: *Firstly, it was reasoned that (any) effects of prior probability on the nondecision time parameter was reflective of effects on the response execution time, and not the stimulus encoding time, component. Given the identical optical properties of the stimuli in the current study (see 'Experimental procedure' section) and that stimulus encoding extracts decision-relevant evidence, it is highly unlikely that the stimulus encoding time component is different for the left vs. right accumulators.*

The sentence formation causes a confusion as to whether the prior probability on the nondecision time parameter is reflective only of reaction time and not response encoding. In the study, the manipulation is on the reaction time so it should be stated more clearly.

- *Page7: ('prime'), regardless of its validity, prior to the imperative 137 signal ('target') might invoke response priming with the visual similarity between the warning and imperative signal possibly invoking perceptual priming (see Fig 2 and 'Experimental procedure' section). Response priming in the current study was tested with the presence of 'catch' trials (i.e., trials where the warning signal was not followed by an imperative signal) such that a negligible influence of response priming would be reflected by participants correctly withholding their responses on catch trials. Perceptual priming was tested by ensuring that the warning signal prime and the imperative signal target utilized the same symbol (arrows as per Fig 2), with any perceptual priming effects biasing sensory evidence and being reflected as superior model fits of consistent evidence-accumulation parameters (v parameter in Fig 1).*

The false alarm rate for catch trials? The sentences are not very clear in this section. It would be good to clarify how response priming and perceptual priming is particularly tested and why.The paradigm is explained nicely in the methods part but it confuses the reader here.

Methods:

Line 347 prediciton -> prediction

Discussion:

- *Line 540 In contrast, previous reports that prior probability affects the quality of sensory evidence found that the more likely outcome has a higher rate of evidence-accumulation and the less likely outcome a lower rate [9,12]. A potential explanation of this discrepancy is that these studies not only required more difficult perceptual discriminations than that required in our study but also utilised a different evidence-accumulation model (drift diffusion model vs. the racing diffusion model utilized here).*

The first sentence should be reformulated. It is very hard to understand. More discussion is needed. Why was racing diffusion model chosen (compared to drift diffusion model for example) in explaining the effects of prior probability?
