# Supplementary material for: What mechanisms mediate prior probability effects on rapid-choice decision-making?
Source: PLoS One. 2023 Jul 7;18(7):e0288085. doi: 10.1371/journal.pone.0288085 (PMC10328325; doi:10.1371/journal.pone.0288085)
Supplement: Attachment — Submitted filename: Response to Reviewers.docx [file pone.0288085.s008.docx]

**Editor**

Thank you for your valuable submission, thoughtful and careful edits.
Please, along with reviewer’s comments, address a few things

a) Double check grammar, there are extensive sections that can be shortened and others that paragraph has two ideas interchangeable at same time.

*The opportunity was taken to double check grammar, shorten sentences, and ensure paragraphs have a consistent flow of ideas.*

b) Check your references accordingly to the Journal’s requirements - keep them constant to help typesetting.

*References have been checked, ensuring they are as per the journal’s requirements.*

c) Remember to detail all stats parameters and reason to pick up them

*This has been detailed and reasoned to the best of our ability.*

Wishing you success with this very good study.

**Reviewer #1**

a) These numbers refer to the appropriate references, in line with PLOS ONE’s Vancouver style referencing guidelines, which utilised block-wise and trialwise biasing procedures, respectively.

After opening manuscript.docx, I finally understood that "('block-wise bias'; 2,3,5,7,8,10,11)" and "('trial-wise bias'; 4,9)" in lines 82-83 are the citation numbers. The peer-review PDF I read had no links to the references, no bracket [], and no explanation of the numbers, so I did not know what the numbers were at first. The Vancouver style recommends brackets [] or superscripts to indicate that the number is a bibliography number. In the published version of the PDF, the numbers are linked to the bibliography, so the effect is minor, but we recommend bracketing the numbers for better clarity. For example, "('block-wise bias'; [2,3,5,7,8,10,11])".

*The references now have square brackets for additional clarity.*

b) We thank the reviewer for the positive feedback about our methodology to ensure fast guesses were excluded from analyses.

I thought this methodology was a good example of analyzing reaction times and one of the most commendable aspects of the paper.

*We thank the reviewer for the positive feedback.*

c) The equation has now been added to manuscript on Page 12, Line 277-284, and reads as, “For example, if the rates for left and right accumulator are vleft and vright before the addition of bias, and denoting the minimum of these two values as dv (typically the rate for the mismatching accumulator), the rates after the addition of bias (with subscripts “right,bias” and “left,bias”) become:
>vright,bias = vright + dv * vbr (a)
>vleft,bias = vleft – dv * vbr (b)
>Note that scaling vbr by dv ensures that the rates remain non-negative.”
>Furthermore, in the spirit of open science, besides the description and equation in the manuscript, the source data and analyses code with model specifications have been uploaded to the Open Science Framework.

Regarding the Model fitting section of the Racing Diffusion Model, this modification makes it easier to understand how the bias effect parameter vbr is specifically implemented, since it is now shown in a mathematical expression. I also confirmed that the analysis data and the source code of R can be downloaded; I wonder if the bias effect in parameters other than v (i.e., B, t0) can also be expressed by the same equation? Or is it possible that only the parameter v is treated specially, since the symbols for the bias parameters for B and t0 are not mentioned in any of the following explanations. A word of explanation on this point would be appreciated.
However, the bias effect parameter explanation should be written more clearly as it confuses the reader. From lines 325-328, I can read that two bias effect parameters are added, and each parameter express that the warning signal is left or right. Since the Btv0 model has 12 parameters, I can enumerate all the parameters of the Bvt0 model, that is, (B_l, B_r, v_l, v_r, t0_l, t0_r, Bbr_l, Bbr_r, vbr_l, vbr_r, t0br_l, t0br_r), where the variables with suffix "br_x" are eliminated in the degenerate model. Here, "_l, _r" are suffices indicating the direction of response or the left and right sides of the accumulator, and "br_l, br_r" are suffices indicating the bias effect when the warning signal is to the left and to the right, respectively. On the other hand, I understood that the formula added in lines 277-284 has only one bias effect parameter, vbr (dv is not a free parameter since it selects the smaller of vright and vleft, and that the common variable vbr is used for v(left,bias) and v(right,bias)). The latter explanation seems to express the left and right bias directions by the sign of vbr and the Sampling section (lines 297-300) also describes only vbr as a bias effect parameter. Therefore, it is seemingly contradictory.

*It is only the v parameter that is treated according to the mathematical expression. For the B and t0 parameters, bias effects for each accumulator are as follows using the reviewer’s notation,*

*Bbr_l, Bbr_r, Bbl_l, Bbl_r, t0br_l, t0br_r, t0bl_l, and t0_bl_r (8 parameters)*

*This is clarified in the manuscript and reads as,*

*“Threshold and nondecision time were parameterised as b and t_0_, respectively, for every bias direction (left and right) and accumulator (left and right) combination.”*

*For v, the 4 parameters are v.true (matching accumulator), v.false (mismatching accumulator), vbr_l (vBR parameter for the leftward pointing warning signal), and vbr_r (vBR parameter for the rightward pointing warning signal). Thus, as the reviewer pointed out, vbr_l would be expected to be negative and vbr_r would be expected to be positive.*

*All parameters for all the models are stated in the ‘model_create.R’ file.*

d) We would like to clarify that the influence of the parameters is considered by removing the bias effect on the parameter, and not the parameter itself. That is, the Bvt0 model where the bias effect of one parameter is omitted (by way of averaging or setting to 0), the Bvt0 model still includes all parameters. This ‘reduced model’ is then compared to the Bvt0 model where the bias effect of all parameters is included (‘full model’). Thus, there are no differences in parameters between the reduced and the full model and the Bt0 model is not the most reasonable purely in comparison to an overfitting Bvt0 model. This is stated on Page 15, Line 345-352, and reads as, ...(omitted below)

To calculate the ratio of bias effects, the author uses a "reduced model" (e.g., the Bt0 model) in which certain parameters are modeled as mean values (or 0) independent of the bias direction (not being removed), and a "full model" (Btv0 model) in which all three parameters are modeled as variables dependent on the bias direction, where all three parameters are modeled as variables that depend on the bias direction. The ratio of the bias effect is the bias effect of the reduced model divided by the bias effect of the full model. Since both models are complete in terms of the parameter types themselves, indeed, this ratio is an indicator of the relative importance of the parameters in the Btv0 model. It is still questionable whether the overfitting Btv0 model can be used as the basis for the indicator, but it is only one of the indicators in this paper. This issue is not critical since the decision is made in combination with other indicators such as the DIC, which calculates the complexity of the model as a cost.

*Besides the use of other indicators, such as the DIC, we must clarify that the ‘reduced’ and ‘full’ models are not the Bt0 model with certain parameters modelled as mean values or 0 and the Bvt0 model in which all three parameters are modelled to be dependent on bias direction, respectively. The ‘full’ models are the Bvt0 model and the Bt0 model where the parameters vary by bias direction whereas the ‘reduced’ models are where the parameters are modelled as mean values or 0. This is presented separately for the Bvt0 model (Table 2) and the Bt0 model (Table 3) in the Results section of the manuscript.*

c) The reviewer makes a good suggestion regarding Inhibition of Return (IOR) underlying the negative evidence accumulation parameters. However, the observed data in Figure 3, as highlighted by the reviewer, does not provide support for this. Specifically, reaction time is not slower for congruent trials (where inhibition of return would be most prevalent) with this pattern bserved in both bias types (block-wise and trial-wise) as well as both age groups (young and older adults). Thus, from our perspective, overfitting is the most likely explanation of the negative bias effects in the Bvt0 model (as stated on Page 19, Line 392).

The author's argument is valid. If there is an effect of inhibition of return (IOR), the reaction time should clearly slow down even in the matching condition. However, Figure 3, which is close to the raw data, shows that the reaction time actually consistently speeded up in the matching direction. This makes it difficult to believe that IOR was occurring and that it was correctly modeled by the Bvt0 model.

*We thank the reviewer for their confirmation of our response.*

d) To ensure the relevant data was highlighted to the highest degree, the choice was made to not overcrowd the figures with additional text via in-figure legends and to instead have all the relevant information in the figure captions.

Yes, I understand. I prefer to have the plot legend in the figure image, but the PLOS ONE guidelines allow authors to decide whether to include the legend in the figure or not. The text is clearly visible by referring to the original tif file, so there is no problem here.

*We thank the reviewer for their confirmation of our response.*

e) The caption of Table 5 has now been amended in line with the reviewer’s feedback with the addition of the below passage on Page 27 (Line 498-501), “For block- and trial-wise bias types, positive values indicate a larger incongruent, than congruent, value, whereas for block-wise vs. trial-wise bias type and young vs. older adult comparisons, positive values indicate a greater bias effect in the former.”

Thanks for the additional passage that made it easier to understand.

*We thank the reviewer for their appreciation of our response.*

f) The reviewer makes a valid point about the number of spikes, and this has now been added to our speculation (Page 29, Line 543), which reads as, “… by requiring more motoneurons and/or greater firing rates…”

Thank you very much for reflecting my point.

*We thank the reviewer for their appreciation of our response.*

**Reviewer #2**

The authors responded all comments satisfactorily. The language was clear, and the data analysis and results were explained clearly.
I thank the authors for their hard work.

I have no further comments or suggestions.

*We are pleased that our responses were to the reviewer’s satisfaction.*
